# Combined Swelling and Metal Infiltration: Advancing Block Copolymer Pattern Control for Nanopatterning Applications

**DOI:** 10.1021/acsanm.4c06197

**Published:** 2025-01-20

**Authors:** Eleanor Mullen, Alberto Alvarez-Fernandez, Nadezda Prochukhan, Arantxa Davó-Quiñonero, Raman Bekarevich, Farzan Gity, Brendan Sheehan, Jhonattan Frank Baez Vasquez, Riley Gatensby, Ahmed Bentaleb, Alan Ward, Paul K. Hurley, Michael A. Morris

**Affiliations:** † Centre for Research on Adaptive Nanostructures and Nanodevices (CRANN) and Advanced Materials and Bioengineering Research (AMBER), Trinity College Dublin, Dublin 2 D02 W085, Ireland; ‡ Centro de Física de Materiales (CFM) (CSIC−UPV/EHU)—Materials Physics Center (MPC), Paseo Manuel de Lardizabal 5, 20018 San Sebastián, Spain; § Inorganic Chemistry Department, University of Alicante, Carretera San Vicente del Raspeig s/n, E-03080 Alicante, Spain; ∥ Advanced Microscopy Laboratory (AML), Centre for Research on Adaptive Nanostructures and Nanodevices (CRANN), Trinity College Dublin, Dublin 2 D02 DA31, Ireland; ⊥ Tyndall National Institute, University College Cork, Lee Maltings, Cork T12 R5CP, Ireland; # Centre de Recherche Paul Pascal (CRPP)—UMR 5031, Pessac 33600, France; ∇ Imperial College London, South Kensington Campus, London SW7 2AZ, United Kingdom

**Keywords:** block copolymer patterning, solvent swelling, vapor-phase infiltration, semiconductor industry, vapor-phase patterning, nanofabrication, nanotechnology

## Abstract

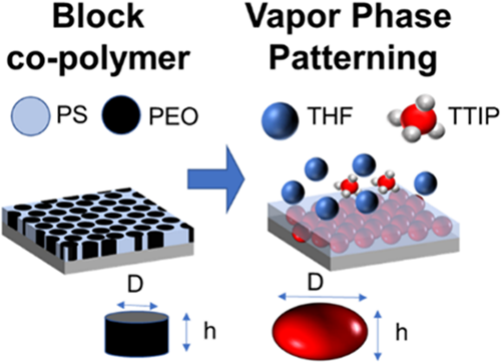

Block copolymer (BCP) patterning is a well-established self-assembly technique for developing surfaces with regular and controllable nanosized features. This method relies on the microphase separation of a BCP film and subsequent infiltration with inorganic species. The BCP film serves as a template, leaving behind inorganic replicas when removed. BCP patterning offers a promising, cost-effective alternative to standard nanopatterning techniques, featuring fewer processing steps and reduced energy use. However, BCP patterning can be complex and challenging to control. Varying the structural characteristics of the polymeric template (feature sizes) requires careful and often challenging synthesis of bespoke BCPs with controllable molecular weights (*M*_w_). To develop BCP patterning as a standard nanofabrication approach, a vapor-phase patterning (VPP) technology has been developed. VPP allows for the *simultaneous, single-step, selective swelling* of BCP nanodomains to precise feature sizes and morphologies while forming inorganic features by metallic precursor infiltration. Infiltration preserves the swollen arrangement, thus allowing for feature size selection without synthesizing BCPs with different *M*_w_, simplifying the process. VPP has the potential to revolutionize nanopatterning techniques in industries such as optical materials, materials for energy storage, sensors, and semiconductors by providing a pathway to efficient, precise, and cost-effective BCP template patterning.

## 1 Introduction

The search for environmentally benign, more efficient, and faster nanofabrication processes has increased interest in block copolymer (BCP) lithography and nanopatterning. BCP template patterning offers an alternative fabrication approach for various applications, including optical materials,^1−3^ capacitors,^4^ separation membranes,^5^ antifouling coatings,^6,7^ integrated circuits,^8^ and Internet of Things (IoT) hardware components.^9^ In particular, the semiconductor industry’s demand for smaller, denser, and more efficient electronic components has driven a continuous search for economically feasible and more sustainable miniaturization of the internal chip components beyond current immersion lithography, double patterning, and extreme ultraviolet technologies.^10^ BCP patterning may offer a versatile, economically favorable, and time-efficient option for reducing the material and energy use of nanopatterning processes.^10−14^

The chemically incompatible polymer chains covalently joined to form a BCP are key to any BCP process.^15^ This chemical incompatibility induces microphase separation within the film under certain thermodynamic conditions, leading to a self-assembled pattern of repeating nanodomains (spherical, cylindrical, gyroidal, or lamellar). A BCP can be selectively infiltrated with inorganic materials exhibiting a chemical attraction to specific binding sites on one of the polymer chains.^16^ The BCP acts as a template that, when removed, yields inorganic nanostructures.^17^ Engineering the BCP’s overall molecular weight (*M*_w_) allows for precise morphological pitch control (nanodomain size).^8,18^ Self-assembly of BCPs is commonly achieved using solvent vapor annealing (SVA) and thermal annealing.^19,20^ SVA can often develop high-precision structural orientation, local alignment, and long-range ordering of BCP films at lower temperatures and shorter times than other techniques.^21,22^ However, a fundamental limitation is that the BCP film thickness and nanodomain dimensions deswell once the solvent is removed.^19,21,23−25^ Continuous control over the structural parameters is impossible with a standard SVA process.

Thus, BCPs with different macromolecular characteristics have been developed to control nanodomain feature sizes.^1^ For example, the fractionation of polydisperse BCPs by size exclusion chromatography to obtain a small library of different *M*_w_ BCPs.^26^ However, the necessity of synthesizing a specific BCP for each desired nanopaterning application remains a significant barrier preventing the large-scale integration of BCP patterning into nanotechnology production. Other studies use small molecules, such as homopolymers, in the BCP system to selectively swell one of the BCP domains, allowing the specific expansion of the final structures.^27−32^ Recently, a combination of selective swelling via SVA and partial locking of the structure through condensation of inorganic sol–gel precursors present in the BCP hybrid film has been explored.^23^ An alternative approach uses atomic layer deposition (ALD) to control nanodomain feature sizes via the number of cycles of inorganic precursors. This method does not require processing steps, such as inorganic sol–gel preparations, and reduces the number of chemicals needed.

ALD and sequential infiltration synthesis (SIS) involve alternating exposure of BCP films to different precursors in sequential half-cycles.^33−35^ SIS is similar to ALD but uses higher partial pressure and exposure times than ALD, allowing for a higher level of precursor entrapment throughout the BCP film.^35^ A typical process can be outlined as follows: During the first half cycle, a vapor-phase metal precursor binds to available binding sites on the reactive polymer block. This is followed by purging the chamber with inert gas to remove excess precursor vapor. An oxidizing agent then reacts with the infiltrated metal precursor to allow further bonding during the second half cycle. This is then followed by another purging cycle, and the process repeats.^36^ The diameter of nanofeatures produced via BCP patterning can be controlled by the number of ALD or SIS cycles used to impregnate a reactive BCP block with a metal precursor.^37,38^ For example, to produce TiO_2_ nanostructures using SIS or ALD, each alternating precursor cycle increases the diameter of the reactive BCP domain. The first half cycle is responsible for titanium precursor binding. The second half cycle hydrolyzes the bound titanium from the first cycle to facilitate further titanium precursor binding to the hydrolyzed titanium during the next half cycle.^39^ However, this approach has low process efficiency, with 20 nm feature size patterning in the order of hours instead of minutes.^37^ Precursor choice is also limited, as infiltration of bulky side chains is impossible when infiltrating micelles.^37^ To improve the quality of precursor infiltration into polymers during SIS, Ko et al.^40^ demonstrated that using an additional organic solvent as a coreactant during SIS of polymer films induces solvent swelling of the polymer film and improves infiltration rate and fidelity. Although solvent swelling increases the number of chemicals needed compared to traditional ALD or SIS, the time required to produce nanostructures of a select size is reduced. However, this technique has not yet been applied to SIS or ALD of inorganic precursors into BCP films to manipulate the infiltrated nanodomains’ morphology and feature size. Additionally, the process outlined by Ko et al.^40^ requires alternating half-cycles of precursors instead of one single exposure cycle.

An advanced nanopatterning tool, a vapor-phase patterning (VPP) system, was developed to expose BCP films to an organic solvent that swells the BCP film while simultaneously infiltrating it with a metal precursor. During VPP, the BCP is swollen by an organic solvent, causing the polymer chains to uncoil. Uncoiling the chains increases the number of binding sites accessible for inorganic precursor binding. Swelling the film with a solvent also assists the diffusion of molecules through the polymer to the metal coordination sites.^40,41^ Thus, infiltration is less dependent on precursor molecule size. The solvent’s flow rate and temperature, as well as the substrate temperature, can all be used to control the film’s swelling rate.^21,42^ Additionally, the metal precursor’s flow rate controls the rate at which the reactive nanodomain is saturated with metal precursor. The metal precursor infiltrates the film, binding to newly accessible binding sites, preventing or reducing deswelling. This is because metal infiltration increases the effective excluded volume of a polymer chain.^43^ Other authors have outlined the use of metal precursors to retain nanofeature structure post-BCP swelling but have never successfully integrated metal infiltration with solvent swelling in a single-step process to select different feature sizes of BCPs.^41^ The potential applications of VPP are vast, as nanopatterning is essential to industries that are fundamental to human society, including energy, technology, and medicine.^44^

Any BCP template and inorganic precursor combination allowing for inorganic precursor binding to select polymer sites could have been chosen to demonstrate the operational principles of VPP. However, polystyrene-*block*-poly(ethylene oxide) (PS-*b*-PEO) was selected as the BCP template, and Titanium(IV) Isopropoxide (TTIP) as the inorganic precursor to further build upon the work previously conducted by Giraud et al.^17^ By using the same polymer and metal precursor, the effectiveness of VPP can be directly compared to the work of Giraud et al.^17^ Their work showcases the successful formation of TiO_2_ nanostructures via a vapor inclusion method.^17^ Similar to this study, vapor-phase infiltration (VPI) of self-assembled PS-*b*-PEO films was achieved by exposing the BCP template postself-assembly to TTIP. However, a swelling solvent was introduced in this study, and the static infiltration chamber was replaced with a dynamic flow system.

During VPP, the domain size and morphology of the nanodomains were altered by varying the exposure time of the BCP to the swelling solvent and metal precursor. Removing the organic BCP template and oxidation of TTIP formed TiO_2_ nanodots of varying sizes dependent upon the VPP time. VPP has enabled tunable sizes and morphologies of nanodomains using a single *M*_w_ BCP and without alternating cycles of precursors. This enables an increased rate of metal binding as compared to SIS or ALD, where the rate-limiting step is the availability of binding sites for each cycle. An increased rate of metal binding facilitates rapid feature size selection, increasing process efficiency. This minimizes power consumption time, crucial for advancing nanotechnology in today’s environmental context. Additionally, VPP having a reduced process time offers a route to reduced material consumption if green chemistry principles are applied to optimize flow rates to be as low as possible without losing functionality. The extension of VPP technology to selectively deposit material without using lithographic masks could hold a promising technological advancement for future semiconductors and other devices.

## 2 Experimental section

### 2.1 Materials

***Polymers:*** PS-*b*-PEO was purchased and used without purification from Polymer Source Inc. Based on existing studies, such as those by Giraud et al.,^17^ a number-average molecular weight (*M*_n_) of *M*_n(PS)_ = 42 kg mol^–1^ and *M*_n(PEO)_ = 11.5 kg mol^–1^ was selected. ***Metal precursor:*** TTIP, 97% purchased from Sigma-Aldrich and used as received. ***Solvents:*** Tetrahydrofuran (THF) (inhibitor-free), toluene (HPLC grade, 99.9%) and acetone (HPLC grade, ≥99.8%) were purchased from Sigma-Aldrich and used as received. ***Substrate*:** Si(100) wafers with a native oxide thickness ∼2–5 nm were used as substrates.

### 2.2 Experimental Methods

#### 2.2.1 Polymer Deposition

Solutions of PS-*b*-PEO were made in toluene (1 wt %). The solutions were left to stir for a minimum of 1 h. The silicon substrates were cleaned first in acetone and then in toluene using ultrasonication for 20 min each. Postcleaning, the silicon wafers were dried using a stream of nitrogen. The 1 wt % BCP solution was then deposited onto the substrate using a SCS G3P-8 spin-coater system at 3000 rpm for 30 s, (25 s deposition with a ramp of 5 s).

#### 2.2.2 SVA

The as-cast BCP films were placed in 150 mL SVA jars and sealed, but the caps were not screwed on tightly to avoid oversaturation. Each jar contained 3 mL of toluene in a small vial and one coated substrate positioned at a 45° angle against the vial. The jars were then put into an oven preheated to 50 °C for 2 h. This procedure for PS-*b*-PEO self-assembly was adapted from the work of Giraud et al.^17^

#### 2.2.3 Dynamic Precursor Infiltration

**Figure 1:**
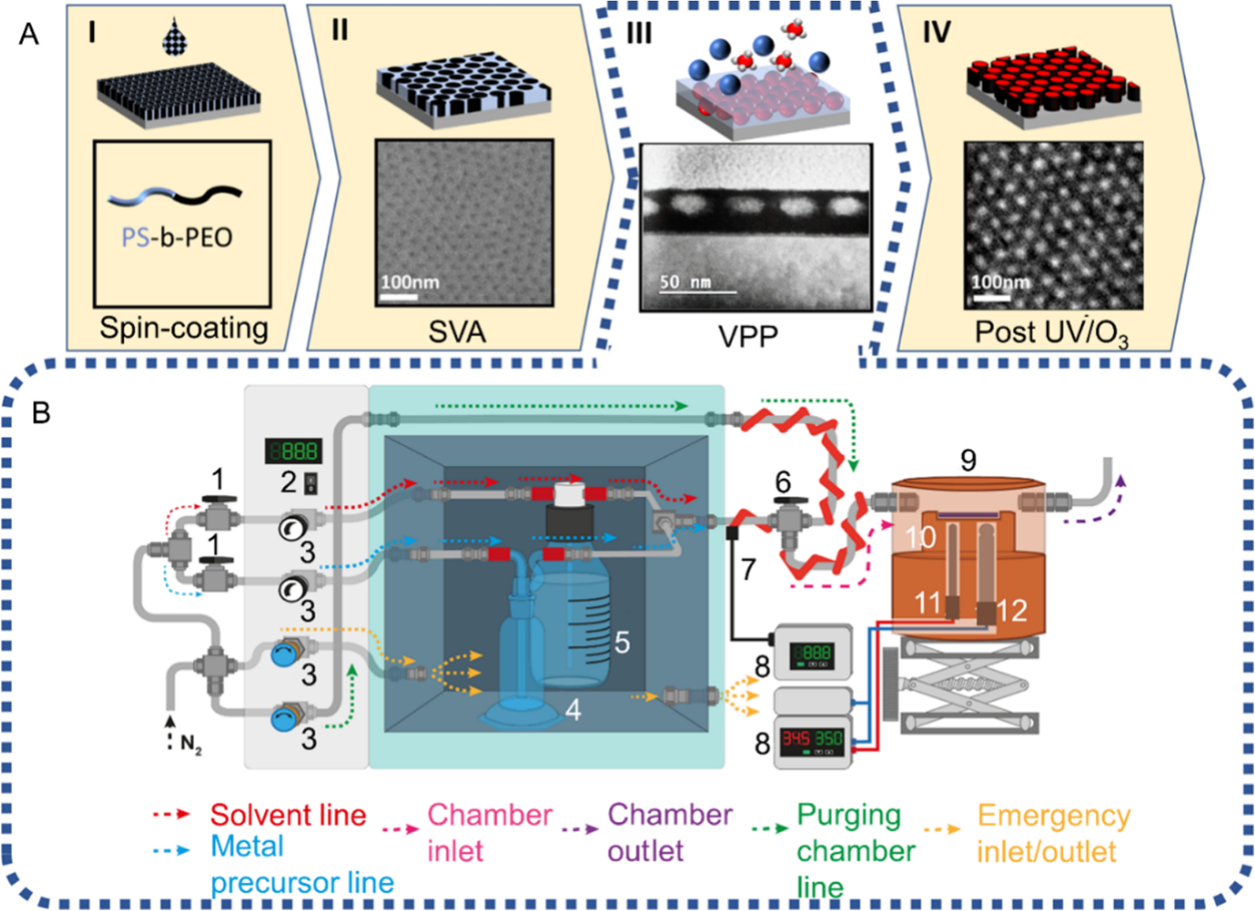
A—controlled BCP patterning process consisting of four nanofabrication stages: (I) the spin coating of PS-*b*-PEO onto a silicon substrate (II) the appearance of the film postself-assembly into vertical cylindrical nanodomains. Inset shows an scanning electron microscopy (SEM) image of the self-assembled structures (III) selection of a specific height and center-to-center distance (*D*_c-c_) for the titanium nanodots in the VPP system. (IV) Post UV ozone treatment. SEM inset demonstrates the resulting nanodots. B—Schematic of the VPP system including details of solvent and metal precursor flow and temperature control: 1—ball valve, 2—Oven Temperature control, 3—Flow metering valve, 4- Metal precursor bubbler, 5—Solvent bubbler, 6—Three-way ball valve, 7—Rope heater, 8—proportional–integral–derivative (PID) controller, 9—Chamber system, 10—Sample stage, 11—resistance temperature detector (RTD) probe and 12—Cartridge heater.

The first stage of using the VPP system for dynamic precursor infiltration is to select the desired operating temperature on the oven, rope heater, and chamber. In this case, a temperature of 35 °C was chosen for all three. TTIP hydrolyzes in the presence of water vapor in the air and forms a white powder. To prevent the hydrolysis of TTIP, the gas lines and bubblers are purged with nitrogen before TTIP is added to the metal precursor line. The solvent and metal precursor bubblers can then be filled with THF and TTIP, respectively. The desired flow rate is then selected on the flow metering valves. The metal and solvent flow metering valves were set to 0.4 L/min in this case. Nitrogen is used as both a carrier gas and to purge the system. The sample was placed in the chamber (Figure 1B9). The chamber is purged for 2–3 min. Following this, the inlet is switched using the three-way ball valve (Figure 1B6) to the mixed metal and solvent vapor line for a set amount of time. In this case, 5, 10, 15, 20, 30, and 70 min intervals were used. Once this time has elapsed, the chamber is purged before sample removal.

#### 2.2.4 Polymer Template Removal via UV Ozone

The deposited titanium hydroxide/dioxide layers were oxidized to form titanium dioxide via 3 h of UV/ozone treatment (PSD Pro Series Digital UV/Ozone System; Novascan Technologies, Inc.). UV/Ozone treatment was also used to ensure complete conversion to TiO_2_ and to remove the polymer template used for the nanopatterning.

#### 2.2.5 In-Situ Ellipsometry Solvent Swelling Studies

A Semilab SE-2000 spectroscopic ellipsometer was used to preform in-situ ellipsometry solvent swelling studies. The system consists of a chamber in which the as-cast BCP film to be swollen sits; the chamber has a viewing port for the ellipsometry analysis, its inlet is connected to a purge line and solvent swelling line, and its outlet to a fume hood (see SI Section S1). Three different studies were performed using this system. The system was purged during these studies after the as-cast BCP film was placed in the chamber and before solvent swelling. For the first study, the solvent vapor (*T*_v_) temperature was set to 18 °C, and the sample temperature (*T*_s_) was set to 22 °C. Solvent swelling was performed using THF and then toluene to demonstrate the effect of solvent choice on BCP swelling. The flow rate for both the first and second study was 0.1 L/min. The second study examined the effect of *T*_s_ on swelling while maintaining a *T*_v_ of 18 °C. The sample stage was heated to 35 °C, and the result was compared to the sample stage at 22 °C. The third study aimed to determine the effect of flow rate on PS-*b*-PEO swelling. This was achieved as follows: the chamber was purged for 5 min, a mass flow controller was used to pass argon gas at 0.1, 0.075, 0.05, and 0.025 L/min into a bubbler containing the chosen solvent, the chamber was then purged again to deswell the BCP film. The change in thickness versus time was monitored using the ellipsometer. The *T*_v_ was set to 18 °C, and *T*_s_ was set to 22 °C. All ellipsometry data analysis was performed with SEA software using Cauchy dispersion model fitting.

### 2.3 Material Characterizations

#### 2.3.1 Scanning Electron Microscopy (SEM)

The surface features of the synthesized samples were visualized by a field-emission scanning electron microscope (FE-SEM) Zeiss Ultra equipped with Everhardt Thornley (SE2) and secondary electron (in-lens) detectors. All images were collected at the previously defined settings,^45^ namely an accelerating voltage of 2 kV for the SE2 detector, 1 kV for the in-lens detector, working distances of 4–5 mm, and 30 μm aperture. ImageJ was used to analyze the feature sizes recorded by SEM imaging.^46^

#### 2.3.2 Focused Ion Beam (FIB)

Sample cross sections (lamella) for analysis using Cross-sectional Transmission Electron Microscopy (XTEM) were prepared using a Dual Beam FIB FEI Helios NanoLab 600i. Several protective layers were formed over the area/surface of interest before FIB milling. Using electron beam-induced deposition, layers consisting of 50–100 nm of carbon and 300–500 nm of platinum were deposited within the DualBeam FIB for all samples with titanium infiltration. When preparing lamella of the polymer template, carbon was not deposited using electron beam-induced deposition as it would give insufficient contrast relative to the PS-*b*-PEO layer during XTEM imaging. Finally, using ion beam-induced deposition, a 3.5 μm thick Carbon layer was deposited on top. The lamellae were prepared and lifted onto a molybdenum sample grid for the XTEM analysis. Lamella thinning for electron transparency was carried out at 30 kV with a final polish at 5 kV (47 pA) to reduce any FIB ion-beam-induced damage.

#### 2.3.3 XTEM

Analysis was performed using an FEI Titan instrument equipped with a Schottky field-emission gun operating at 300 kV. The elemental compositions of the samples were analyzed and mapped within a 10 eV channel using a Bruker XFlash 6–30 Energy dispersive X-ray spectroscopy (EDX) detector with a 30 mm^2^ active area chip and an energy resolution of 129 eV. XTEM images were analyzed using ImageJ (see SI Section S2).^46^

#### 2.3.4 Atomic Force Microscopy (AFM)

AFM Park systems, XE7 was used to examine the quality of PS-*b*-PEO self-assembled templates before use in the VPP system to ensure high-quality pattern templating. The system was operated with a noncontact cantilever (AC160TS, force constant ∼26 N m^–1^, resonant frequency ∼300 kHz) in noncontact adaptive mode. AFM was used to ensure the BCP films were of sufficient quality for vapor-phase infiltration. XEI software (Park Systems Corp.) was used to process the AFM images.

#### 2.3.5 Grazing Incidence Small-Angle X-ray Scattering (GISAXS) Experiments

GISAXS were conducted at the Center de Recherche Paul Pascal (CRPP), Université de Bordeaux, using a high-resolution X-ray spectrometer, Xeuss 2.0 (Xenoxs), operating at a radiation wavelength of λ = 1.54 Å. Two-dimensional (2D) scattering patterns were captured using a PILATUS 300 K Dectris detector positioned at a sample-to-detector distance of 2463 mm. The calibration of the beam center position and the angular range was achieved by employing a silver behenate standard sample. GISAXS data analysis was accomplished with the FitGISAXS software.^47^

#### 2.3.6 Spectroscopy Ellipsometry (SE)

SE was used to record nanodot heights post VPP. Once again, a Semilab SE-2000 spectroscopic ellipsometer was used, and data analysis was performed using SEA software and Cauchy dispersion model fitting.

#### 2.3.7 X-ray Photoelectron Spectroscopy (XPS)

XPS analysis was performed under ultrahigh vacuum conditions (<5 × 10^–9^ mbar) with a nonmonochromated source of Al Kα X-rays (1486.6 eV) operating at 200 W (CTX400, PSP Vacuum Technology). The emitted photoelectrons were collected at a takeoff angle of 90° from the sample surface and analyzed in a RESOLVE120 spectrometer (PSP Vacuum Technology). XPS spectra were recorded by setting the analyzer pass energies constant to 100 and 50 eV, for the survey and core scans, respectively. The peak positions of the photoemission lines were corrected to the C 1s transition at a binding energy of 284.8 eV.^48^

## 3 Results and Discussion

### 3.1 Stages of Nanofabrication

Tunable TiO_2_ nanoarchitectures were obtained following the methodology illustrated in Figure 1. Initially, PS-*b*-PEO/toluene solutions were spin-coated onto a silicon substrate (Figure 1A(I)).^17^ Toluene vapors at 50 °C promoted self-assembly of the BCP film into vertically aligned cylindrical nanodomains (Figure 1A(II)).^17^ Subsequently, different morphologies and feature sizes were formed from a single self-assembled BCP template without requiring an additional SVA step or changing the *M*_w_ of the polymer (Figure 1A(III)). This was achieved using the VPP system (Figure 1B), where a self-assembled BCP film is swollen by suitable solvent exposure and infiltrated with the TiO_2_ precursor. The TiO_2_ nanostructures were obtained after removing the BCP by exposing the hybrid BCP/inorganic precursor samples to UV ozone for 3 h (Figure 1A(IV)).

### 3.2 VPP System Setup

A bespoke VPP system was designed to precisely regulate the kinetics of the metal precursor and swelling solvents’ interaction with the BCP film, enabling the attainment of different nanofeature sizes and morphologies from a single BCP template. This was achieved by precisely controlling parameters such as temperature, flow rate, metal precursor and solvent concentration ratio during feature size selection, exposure time, pressure, and solvent and metal precursor selection. A schematic of the VPP setup is shown in Figure 1B. Rigid stainless steel gas lines were used throughout. The solvent for the BCP swelling (Figure 1B(5)) and metal precursor for the metal infiltration (Figure 1B(4)) are contained within bubblers inside an oven. The metal precursor and solvent vapor pressure are temperature dependent and thus can be altered by changing the temperature of the oven (Figure 1B(2)). The vapor pressure determines the saturation of nitrogen gas with the solvent or metal precursor. The ratio of solvent to metal precursor is controlled by flow metering valves (Figure 1B(3)), which regulate the amount of nitrogen carrier gas entering each bubbler. When the solvent and metal precursors enter the same gas line and mix the temperature is controlled independently by a rope heater (Figure 1B(7)). Following this, the mixed gas line enters a chamber with independent temperature control of the substrate (Figure 1B(9)). The copper stage inside the chamber is heated using a cartridge heater and monitored by an RTD probe connected to a PID controller (Figure 1B(12) and B(11)). All temperature controls can heat the substrate, precursor, or solvent in the range of 0 to 200 °C. The volume inside the chamber holding the sample is as small as possible, thereby maximizing the surface-to-volume ratio for improved infiltration and swelling control.^11^ Thus, the chamber design forces gas flow as close as possible to the sample surface. The chamber is not pressurized or under vacuum but is operated close to atmospheric pressure.

Before VPP can begin, a suitable metal precursor and solvent combination must be selected (See SI Section S3). This study chose TTIP as a model compound and learning vehicle for this new VPP technique. TTIP is suitable because of the high affinity and selectivity of the Ti^4+^ cations for the PEO domains.^17^ Moreover, its high vapor pressure and volatility make TTIP an ideal inorganic precursor for this study.^17,49^ Traces of suitable molecules, such as water, must react with the isopropyl side groups of TTIP to form isopropanol so that the precursor molecules’ titanium (Ti) centers can react with the binding site in the PEO nanodomains.^17,50^ The solvent chosen to control the swelling of PEO must not cause rapid hydrolysis of TTIP and, thus, crystallization in the chamber inlet. After excluding solvents, such as ethanol, which are likely to cause premature crystallization in the pipelines, numerous solvent choices could be trialed; the Flory–Huggins model based on the Hansen solubility parameters was used to identify suitable solvent candidates with high selectivity for PEO, as detailed in SI Section S3. THF was selected for VPP as it is a suitable solvent of PEO (χ_EO-THF_ = 0.27) and is readily available. Additionally, the hygroscopicity of THF means that it can provide low-level traces of water required for TTIP binding to PEO domains.^17,50^

### 3.3 Film Swelling Control

**Figure 2:**
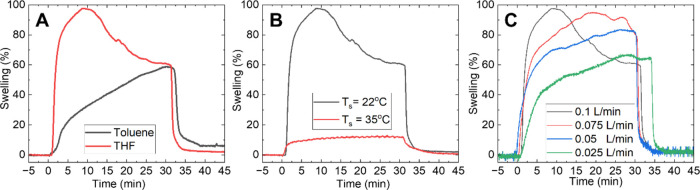
Effect of (A) Toluene and THF solvents on swelling, (B) substrate temperature on THF swelling, and (C) THF flow rate on swelling profiles of PS-*b*-PEO. *T*_v_ denotes the solvent vapor temperature, and *T*_s_ the sample temperature within the solvent swelling chamber.

Solvent swelling studies were conducted to demonstrate how the solvent selected, the effects of temperature, and the solvent’s flow rate impact the swelling of an as-cast BCP film. The aim was not to produce perfectly ordered thin films but to demonstrate the different effects of these parameters on film swelling. The results of the solvent swelling studies are displayed in Figure 2. It is important to note that for all solvent swelling studies performed, it was observed that removing the solvent vapors from BCP films resulted in deswelling.^19,21,24,25^ However, the film sometimes deswelled before chamber purging due to dewetting. Dewetting occurs when sufficient saturation of the film with solvent changes the polymer film from a mixed state to a demixed state.^51^ The BCP film swelling profiles in Figure 2 do not consistently return to a completely unswollen (0% swelling) state after chamber purging within the recorded time frame. This is due to incomplete solvent evaporation from the film, which prevents a complete reduction of the polymer chain’s free volume to its original value.^52^

The impact of THF and toluene on BCP swelling was demonstrated using an ellipsometry solvent swelling chamber capable of recording in situ film thickness changes. Toluene was chosen to illustrate the effect of a less favorable solvent of PEO (χ_EO-Tol_ = 1.91) on swelling. To test the effects of each solvent, an as-cast BCP film was first placed in the chamber, and the system was purged. *T*_v_ was set to 18 °C, and *T*_s_ was set to 22 °C. The select solvent vapor was then flowed into the chamber at 0.1 L/min. After 30 to 35 min of swelling, the system was purged, resulting in the deswelling of the film. The film was swollen by 98% of the original thickness in the case of THF and by 59% in the case of toluene (Figure 2A). The rate at which the maximum swelling was obtained also differs between the two solvents. See SI Section S4 for the equation used to calculate swelling (%) on the *y*-axis in Figure 2. After 30 min of toluene exposure, the maximum thickness of the BCP film was not yet achieved (Figure 2A). The lower vapor pressure of toluene and larger Florry-Huggins parameter (χ) value compared to THF explains its slower swelling and lower swelling ratio (Table S1).^23^ Please refer to SI Section S4 for further details regarding film order post-SVA.

The ellipsometric system was used next to demonstrate how the relative difference between *T*_v_ and *T*_s_ affects solvent swelling profiles. *T*_v_ was held at a constant 18 °C, and *T*_s_ was set first to 22 °C (to test the effect of a few degrees difference between *T*_v_ and *T*_s_) and then 35 °C (SI Section S5 for details on temperature choice). THF was selected as the swelling solvent and entered the chamber for both choices of *T*_s_ at a rate of 0.1 L/min. Figure 2B shows that if *T*_s_ is closer to *T*_v_ as is the case when *T*_s_ is 22 °C and *T*_v_ 18 °C, the relative saturation of the solvent vapor is higher, leading to greater swelling. If *T*_s_ is increased to 35 °C, steady-state swelling is observed in Figure 2B. However, this reduces the relative saturation of the solvent vapor, leading to reduced swelling. If *T*_s_ is much greater than *T*_v_, this reduces solvent uptake and reduces the film’s swelling ratio.^14,21^ However, if *T*_s_ is less than *T*_v_, excessive solvent condensation onto the polymer film may cause film rupture or deswelling.^53^ Thus, the ideal *T*_s_ is slightly greater than or equal to *T*_v_ for rapid swelling.

Vapor flow rate is another critical parameter to consider during the swelling process. To study this, the swelling profiles of PS-*b*-PEO films with THF at flow rates of 0.1, 0.075, 0.05, and 0.025 L/min were recorded (Figure 2C) using the same system as before. *T*_v_ was set to 18 °C, and *T*_s_ was set to 22 °C. The gas kinetics in the chamber depend on the chosen solvent’s flow rate (in this case, THF) into the chamber. At a flow rate of 0.1 L/min, the maximum swelling ratio is reached in less than 10 min, while for reduced flow rates, it is only reached at the end of the SVA treatment. The maximum swelling ratio reached is 3% higher for 0.1 L/min compared to 0.075 L/min, 14% higher for 0.1 L/min compared to 0.05 L/min, and 30% higher for 0.1 L/min compared to 0.025 L/min. The saturation rate of the chamber volume with vapors, in combination with gas kinetics, affects the time taken to achieve maximum swelling and the maximum swelling ratio reached. The film remains disordered after swelling regardless of solvent flow rate.

### 3.4 Simultaneous Metal Infiltration and Solvent Swelling

VPP uses solvent swelling to increase the PEO domains’ size while simultaneously infiltrating them with the TTIP precursor. As demonstrated in the previous section, solvent temperature, flow rate, exposure time, and solvent choice determine the effect of solvent swelling on the films’ morphology. Thus, the results of the previous section highlight the importance of carefully selecting these parameters for VPP. During VPP, metal infiltration prevents rapid film deswelling when solvent saturation occurs or solvent swelling is stopped. When the PEO domains are swollen, the number of accessible binding sites available for TTIP binding increases; thus, swelling the PEO domains allows for a rapid rise in feature sizes. The balance between solvent swelling and metal precursor diffusion and entrapment determines the growth rate of the nanodomains. If the growth rate is fixed, VPP exposure time can be adjusted to obtain the required feature sizes and morphology. This study used the exposure time of solvent swelling and metal infiltration into previously self-assembled BCP films to select different pre-UV ozone film morphologies and post-UV ozone nanodot height and diameter while keeping all other variables constant. Time is chosen as the primary variable of interest, but future studies will examine the effect of holding time constant and varying other variables.

Before any experiments were conducted, a suitable starting point for all the experimental constants had to be selected. The temperature of the sample stage and solvent/metal vapors was set at 35 °C to allow a suitable partial vapor pressure of the solvents and to prevent condensation of the metal precursor in the gas lines (SI Section S5). Having *T*_s_ and *T*_v_ close together, as observed in the previous section, has the added advantage of increased solvent vapor saturation and, thus, increased film swelling rate. The flow rate was approximately 0.4 L/min (four times higher than the highest flow rate in the swelling studies). The aim of increasing the flow rate and having a highly saturated solvent vapor was to achieve almost instantaneous swelling of the polymer template while simultaneously infiltrating with a metal precursor to retain the pattern structure. As discussed in the introduction, solvent swelling increases diffusion into the film, improving the rate of metal infiltration and, thus, feature size selection. This maximizes the range of feature sizes achieved in a smaller VPP time, thus improving process efficiency. Achieving a highly efficient process is a key requirement for the industrial incorporation of polymer-based nanopatterning techniques.^14^ Furthermore, The temperature of the solvent and metal precursor relative to the substrate was not varied because the effect of temperature gradients on VPP was not the variable of interest. Similarly, an equal flow rate of THF and TTIP entering the chamber was set. Any variation among flow variables may influence the resulting nanopatterned features, potentially confounding the primary variable of interest: time. Once all the experimental parameters were set, self-assembled BCP thin films were introduced to the VPP chamber and exposed to different swelling/infiltration times (5, 10, 15, 20, 30, and 75 min).

### 3.5 Morphology

**Figure 3:**
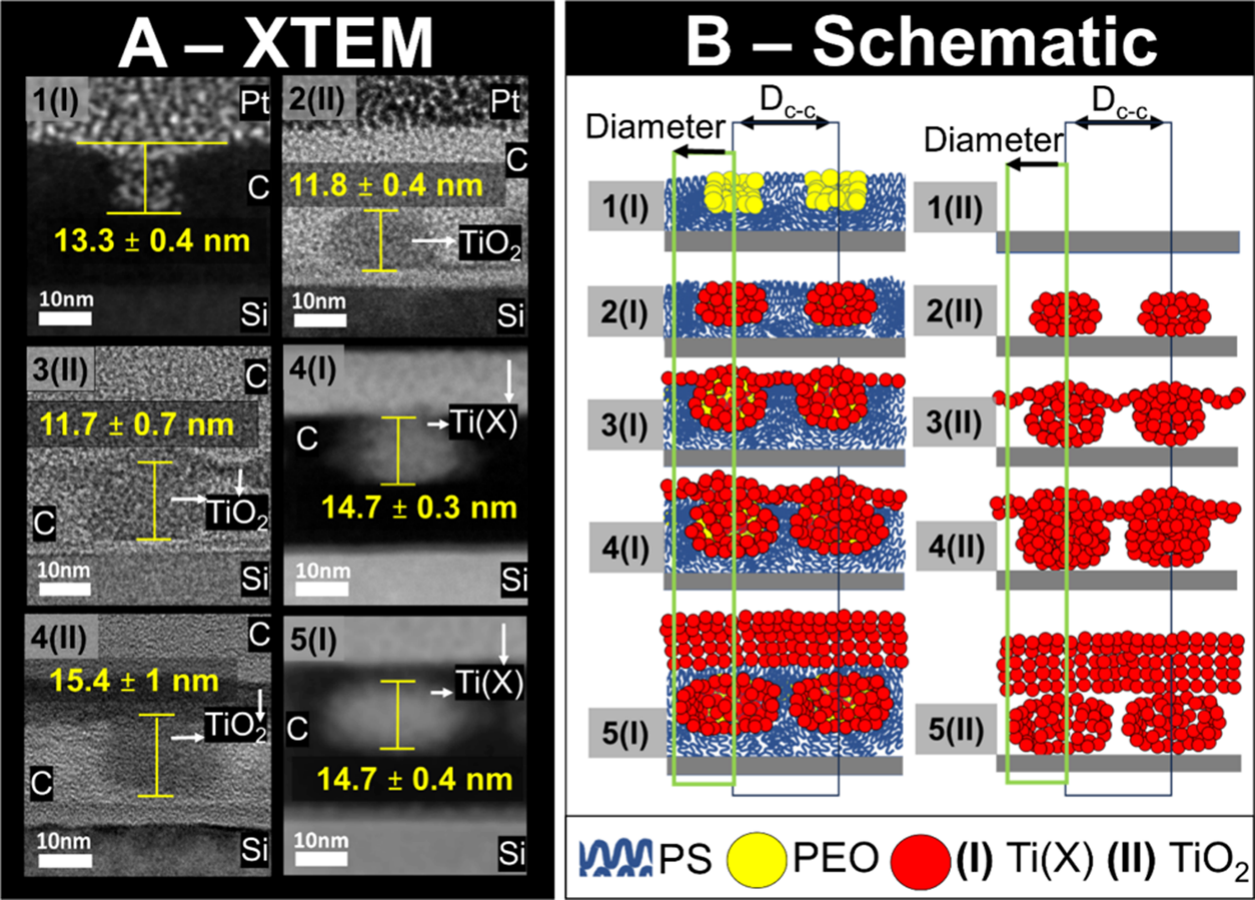
(A) XTEM images reporting nanodomain or nanodot height and associated standard error of the mean. (B) Schematic of TTIP infiltration into PEO domains and nanodot formation. (I) denoted Pre-UV Ozone and (II) Post-UV Ozone. Infiltration times are denoted numerically as follows: (1) 0 min, (2) 5 min, (3) 10 min, (4) 15 min, and (5) 30 min. XTEM images are used to construct the schematic. Schematics are based on the results from XTEM, SEM, and GISAXS.

To study the effect of the swelling/infiltration time on the height of the obtained TiO_2_ nanodots, XTEM images were obtained for different exposure times of 5, 10, 15, 20, and 30 min (Figure 3A). The XTEM results were used to produce schematics illustrating the process of titanium infiltration into the PEO domains pre-UV ozone (Figure 3B subsets 1–5(I)) and the resulting nanodots post-UV ozone (Figure 3B subsets 1–5(II)). ImageJ software was used to obtain the feature sizes of the nanodots (overview of XTEM ImageJ analysis SI Section S2). The average height of the dots for each swelling/infiltration time is shown on the XTEM images presented in Figure 3A. Error analysis for all height measurements is included in SI Section S6. EDX maps were used to distinguish the layers of material corresponding to different elements. The layers of material shown in the XTEM images in Figure 3A were then labeled using the EDX maps in SI Section S7.

The effect of VPP on self-assembled nanodomains was investigated to see if there were any changes in the self-assembled pattern with higher VPP times. The XTEM of the self-assembled PS-*b*-PEO template is shown in Figure 3A subset 1(I). The SVA process generated a PS preferentially wetted substrate with vertically aligned well-ordered cylindrical PEO nanodomains within a PS matrix above. Metal nanodots appear elliptical in shape post-UV ozone XTEM images for 5 and 10 min VPP times (Figure 3A subset 2(II) and 3A subset 3(II)). It is hypothesized that inorganic precursor infiltration into the PEO domains and the greater affinity THF has for swelling the PEO domains leads to a higher swelling rate than that of the PS domains (SI Section S3). This effectively compresses the space PS domains occupy between the PEO domains, resulting in the upward expansion of the PS domains and partial encapsulation of the PEO domains in a PS matrix. This is most evident after a VPP time of 15 min. Thus, micelles are partially formed, with the top of the PEO domains remaining connected to the film’s surface (Figure 3A subset 4(I)). When the solvent and metal saturation reaches a critical value, the chain extension in the PS matrix encapsulates the PEO domain. This is visible at a VPP time of 30 min when there is a clear evolution from cylindrical nanodomains to micellar nanodomains disconnected from the film surface. (Figure 3A subset 5(I)). After 30 min a PS layer formed between the metal film and the nanodot of approximately 2.5 ± 0.3 nm. This result suggests that VPP can transition from perpendicularly aligned cylinders to micelles.

Post-UV ozone, the final morphology of the nanodots appears to have higher curvature than the straight edges of a cylinder, depending on what level of transition toward micellar formation was obtained before UV ozone. If further optimized, there may be some practical applications for manipulating the nanodots’ curvature from flat-toped cylinders to more elliptical nanodots with round tops. Furthermore, the transition from cylindrical domains to micelles during VPP results in a PS layer between the nanodots and excess TiO_2_ deposited on top of the BCP film. During selective etching, this PS layer could act as a protective layer for the nanodots.

Post VPP, the self-assembled pattern remains the same as the initial self-assembled BCP film placed in the chamber. Solvent swelling does not, for example, cause flipping between perpendicular and parallel-aligned cylindrical domains, as observed by Mokarian-Tabari et al.^54^ This may be because the TiO_2_ binding reduces film mobility. Alternatively, the substrate temperature may be too low to bring about a transition, such as the flipping of morphology from perpendicular to parallel cylinder alignment. Further research could investigate the viability of lower initial metal infiltration and higher swelling to bring about the flipping of morphology observed by Mokarian-Tabari et al.^54^ before the critical point in which metal infiltration reduces PEO mobility.

### 3.6 Effect of VPP Time on Height

Once the PEO sites are saturated, the selectivity of the metal binding is lost, and the nanodots act as nucleation sights for further metal deposition. Metal is then deposited on the nanodots and on top of the PS domains surrounding the nanodots. A 5 min VPP exposure time resulted in largely no deposition of TiO_2_ between nanodots because not all PEO domains were fully saturated; however, some thin lines of metal formed in instances where deposition occurred between the nanodots (SI Section S8). When UV ozone was used to remove the polymer and fully oxidize TTIP to TiO_2_, any TiO_2_ on top of the PS layer between the nanodots collapsed onto the silicon substrate (SI Section S8). The collapse occurred because the TiO_2_ layer was too thin to withstand the removal of the supporting PS layer without detaching from the top of the nanodots.

A 10 and 15 min VPP exposure time resulted in complete saturation of the PEO domains and formation of a metal layer on top of the PS domains pre-UV ozone, which was sufficiently thick to withstand collapse post PS layer removal. However, the layer dipped between nanodot supports, which suspended the TiO_2_ layer above the silicon substrate, as illustrated in Figure 3A subset 3(II) and 4(II). The titanium layer measured post-UV ozone was approximately 4.7 ± 0.2 and 11.1 ± 0.3 nm for 10 and 15 min VPP times, respectively (SI Section S6). The TiO_2_ layer increases the perceived height of the nanodots by depositing on top of them. However, as the VPP time increases, the shape of the nanodots is lost beneath progressively more TiO_2_ layers, which smooth out and effectively average the surface, thereby concealing the nanodots. After 30 min, the TiO_2_ layer post UV ozone is 43 ± 0.1 nm (SI Section S6). SEM analysis cannot measure the diameter of nanodots produced from VPP times of 30 min as the nanodots are beneath too thick a layer of TiO_2_.

The PEO domains post BCP SVA are approximately 13.3 ± 0.4 nm in height (Figure 3A subset 1(I)). A 5 min VPP process results in nanodots of height approximately 11.8 ± 0.4 nm (Figure 3A subset 2(II)). The nanodot height in the case of a 10 min process is approximately 11.7 ± 0.7 nm (Figure 3A subset 3(II)). In the case of the 15 min infiltration, nanodot heights are 14.7 ± 0.3 nm pre-UV ozone and 15.4 ± 1 nm post-UV ozone (Figure 3A subset 4(I) and 4(II)). This suggests no increase of nanodot height beyond the original template for VPP times less than 15 min. The difference measured between the pre- and post-UV ozone nanodot height is less than the image resolution, so we can assume UV ozone does not impact nanodot height. The height of nanodots post-UV ozone in the case of a 30 min infiltration time is 14.7 nm ±0.4 nm (approximately equal to a 15 min VPP time) (Figure 3A subset 5(I)). We conclude that with increasing VPP exposure time, nanodot height increases from an initial thickness of approximately 11.8 nm to approximately 14.7 nm.

**Figure 4:**
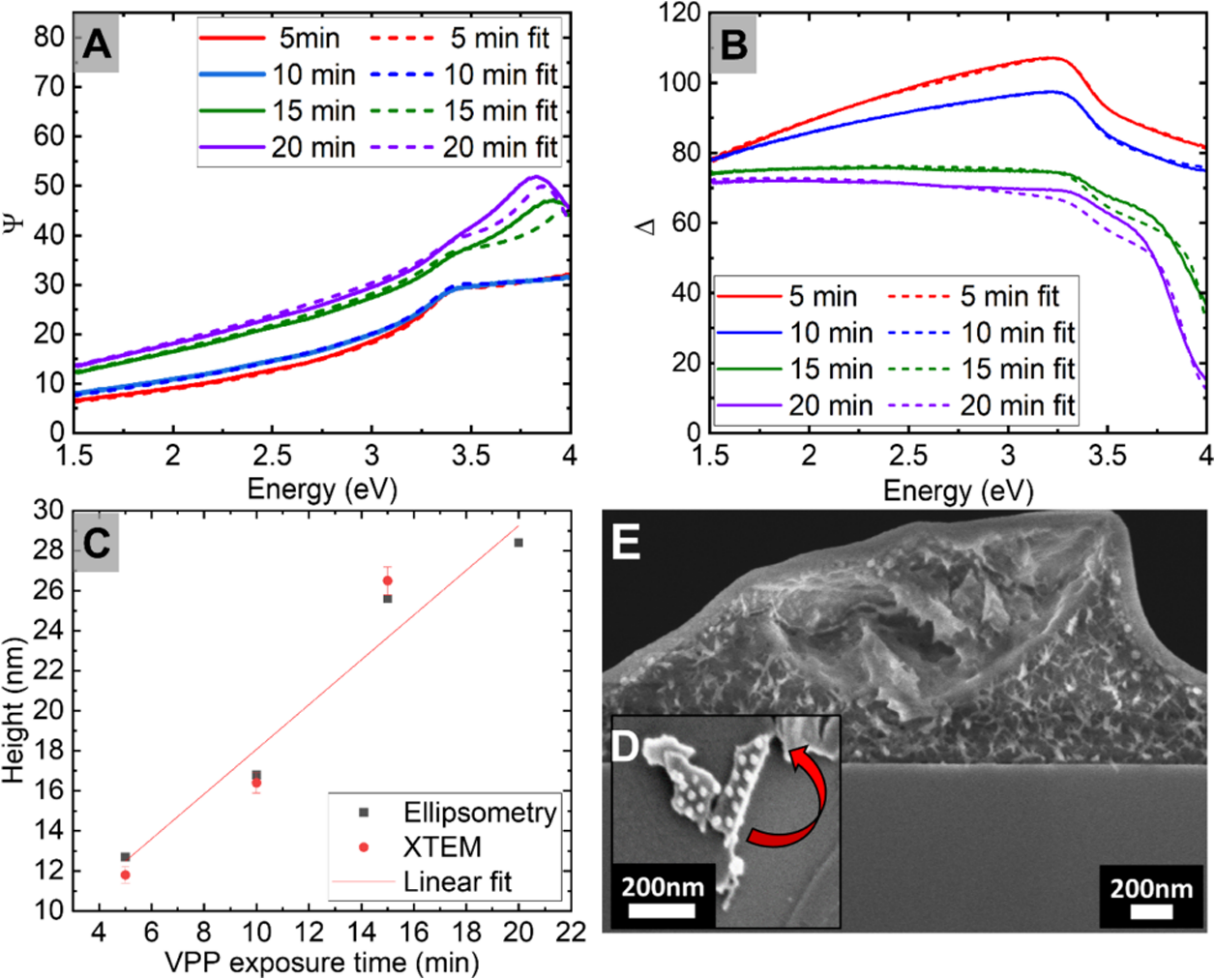
Spectroscopic ellipsometry data (A) Psi versus energy and (B) Delta versus energy. (C) A graph of nanodot heights recorded by ellipsometry and XTEM versus VPP exposure time (error bars report the magnitude of the standard error of the mean). (D) Pealing up of post-UV ozone film to expose nanodots. (E) Bending of titanium-polymer composite film pre-UV ozone.

SE allows for the scanning of larger 2–3 mm areas. SE was used to support further observations concerning the swelling/infiltration time (Figure 4A,B). XTEM measurements of combined nanodot and TiO_2_ height for the various VPP times post UV Ozone were as follows: 11.8 ± 0.4 nm (5 min), 16.4 ± 0.5 nm (10 min), and 26.5 ± 0.7 nm (15 min) (SI Section S6). Post-UV ozone XTEM and SE results differ by a maximum of ±1.1 nm (Figure 4C and SI Section S9). The slope of the linear fit in Figure 4C is 1.1 ± 0.2; thus, for every 1 min increase in VPP time, the TiO_2_ combined dot and film height increases by approximately 1.1 nm.

In the case of the 15 min VPP sample, the TiO_2_ layer is thick enough for the titanium dots to be completely interconnected through the TiO_2_ layer. The film can be peeled up, as shown in Figure 4D. The nanodots remain bound to the TiO_2_ layer instead of the silicon substrate (Figure 4D). No pattern deformation is observed. The TiO_2_ layers can be peeled up using a sticky surface, exposing the nanodots underneath. Thus, if a sufficiently thick TiO_2_ layer binds the nanodots, post-UV ozone, a TiO_2_ nanodot tape can be created. Alternatively, the nanodot layers could be peeled off, suspended in a solution, and used in paint. Applications of the production of TiO_2_ nanostructures on a TiO_2_ layer include antifouling, antiadhesion, and self-cleaning surfaces.^55^ For high curvature applications, TiO_2_ nanodot tape using the pre-UV ozone film may be more advantageous. This is because the TiO_2_ layer, consisting of a thin film patterned with nanodots, is still bonded to the PS layer, allowing flexibility to bend the titanium layer without the film cracking. Figure 4E shows the bending of a film pre-UV ozone. Once the film is attached to the substrate of high curvature, UV ozone can be used to remove the polymer and fix the film in place. Applications include TiO_2_ nanodot patterning on curved glass for optical sensors.^56^

### 3.7 Effect of VPP Time on Diameter and *D*_c-c_

**Figure 5:**
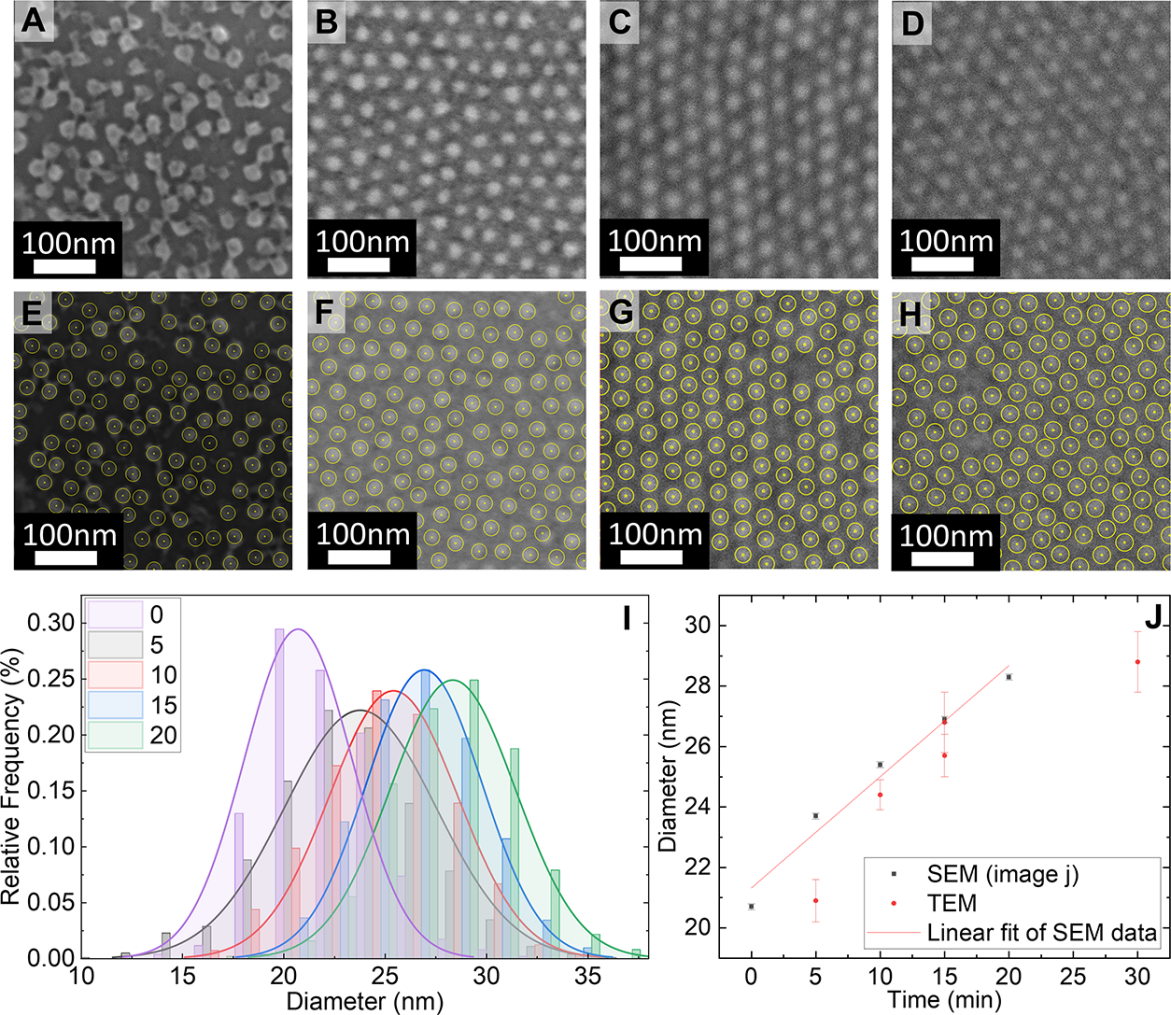
Top-view SEM micrographs of the post-UV ozoneTiO_2_ nanodot obtained after different VPP exposure times: (A) 5 min; (B) 10 min; (C) 15 min; and (D) 20 min. (E–H) Image overlay with circles of average nanodot diameter for (E) 5 min, (F) 10 min, (G) 15 min, and (H) 20 min. The circles contain an inner concentric circle with a diameter approaching zero, which is used to measure *D*_c-c_. (I) Corresponding nanodot diameter histograms obtained by image analysis. (J) The average diameter was recorded for different exposure times using post UV ozone SEM and XTEM analysis of post-UV and pre-UV ozone samples (error bars report the magnitude of the standard error of the mean).

Top-view SEM micrographs of the inorganic replicas obtained after the UV/O_3_ treatment are shown in Figure 5A–D. To gain more insight into the structural changes observed, ImageJ software was used to extract the diameter of the TiO_2_ nanodots (method detailed in SI Section S10).^46^ The nanodot diameters for the different VPP times and corresponding error analysis are provided in SI Section S11. Image overlay with average nanodot diameter recorded for 5, 10, 15, and 20 min are shown in Figure 5E–H. Other measurement techniques were constrained by the need to adjust image contrast for nanodot recognition software to function, which affected the results’ reliability (SI Section S12). From the histograms in Figure 5I, a continuous increase in the diameter of the TiO_2_ nanodots can be observed as VPP time increases. The diameters of the obtained nanofeatures grow with increased swelling/infiltration time from 20.7 ± 0.1 nm (0 min) to 23.7 ± 0.1 nm (5 min), 25.4 ± 0.1 nm (10 min), 26.9 ± 0.1 nm (15 min), and 28.3 ± 0.1 nm (20 min). Despite the slight overlap between the histograms in Figure 5I, the increase in diameter as time increases is statistically significant (SI Section S13).

XTEM micrographs were also used to measure diameter (results included in SI Section S14). The diameter measurements from XTEM and SEM analysis for pre and post-UV ozone were plotted in Figure 5J as they confirm the trend of increasing nanodot diameter. Due to the minimal sample size and the dependence of observed diameters on the angle at which the lamella is taken, these data points were excluded from the linear fit (SI Section S14). The slope of the linear fit is approximately 0.368; thus, for every 1 min increase in VPP time, there is an approximate 0.4 nm increase in diameter.

To investigate the process efficiency of VPP diameter selection, the time it takes to produce TiO_2_ nanostructures of select sizes was compared with other studies that do not use solvent swelling. Giraud et al.,^17^ used self-assembled PS_42k_-*b*-PEO_11.5k_ as a template for TTIP infiltration during a VPI process in a glass vial. A 3-h exposure time was required to generate nanostructures of sizes comparable to the original BCP template postcalcination. This study also uses TTIP as a metal precursor and PS_42k_-*b*-PEO_11.5k_ as a BCP template. However, the VPI process occurs in a VPP system, and THF is used as a swelling solvent during VPI of the PEO nanodomains with TTIP. Nanostructures of sizes comparable to the original PS_42k_-*b*-PEO_11.5k_ template are achieved within 5 min. The transition from a glass vial to a VPP system in which precursors flow over the substrate in a nonstatic system may partly contribute to the increased rate. However, as demonstrated by Ko et al.^40^ and She et al.,^41^ swelling a BCP or polymer film before infiltration or during ALD or SIS increases the rate and quality of inorganic precursor infiltration. Thus, the dramatic increase in infiltration rate cannot be attributed simply to the change in the design of the VPI system, as solvent swelling is detrimental to determining infiltration rates of inorganic precursors into polymers.

To further assess how VPP improves the infiltration rate into BCP templates, the ALD approach to BCP pattern control outlined by Yin et al.^37^ was compared to VPP regarding process efficiency. Yin et al.^37^ infiltrated polystyrene-*block*-poly(4-vinylpyridine) (PS-*b*-P4VP) micelles with alternating cycles of TiCl_4_ and H_2_O. It took approximately 99 cycles (approximately 231 min) to produce TiO_2_ nanodots measuring 28 nm in diameter. Conversely, using VPP took 15 min to produce nanodots of approximately 28 nm in diameter. Yin et al.^37^ used a polymer system that is different from that used in our study. Still, given the difference in time, it is unlikely that changing the precursor or polymer would reduce the process time to be competitive with VPP.

**Figure 6:**
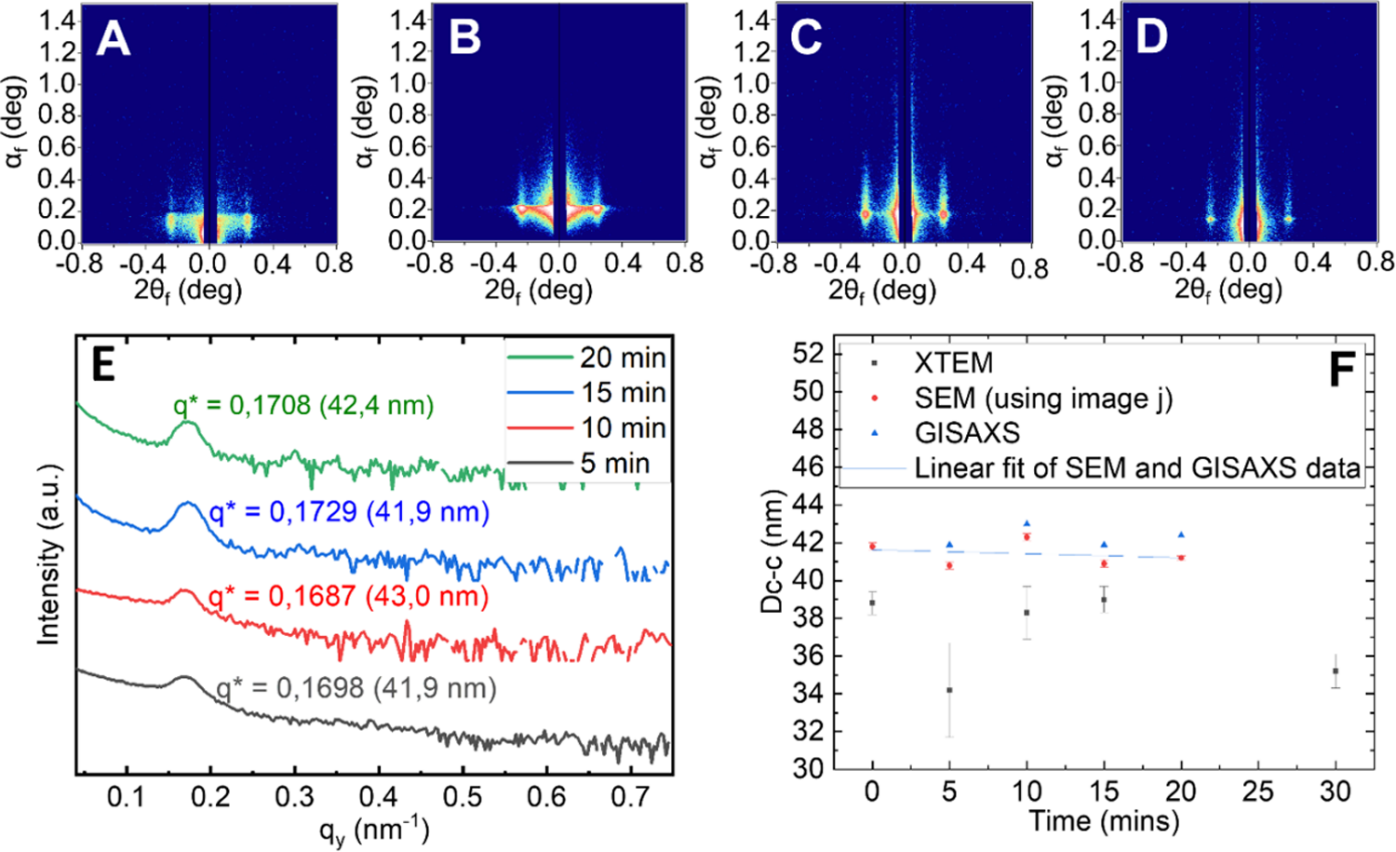
GISAXS 2D scattering patterns of the PS-*b*-PEO films undergoing dual THF swelling and TTIP metal infiltration for set time intervals (A) 5 min, (B) 10 min, (C) 15 min, and (D) 20 min. (E) one-dimensional (1D) Intensity profiles extracted from the SAXS images. (F) Plot comparing measurements from the different characterization techniques for *D*_c-c_ (error bars report the magnitude of the standard error of the mean).

The *D*_c-c_ distance between the TiO_2_ nanodots was measured using ImageJ analysis of the SEM images (method detailed in SI Section S10). All *D*_c-c_ recorded ranged from a minimum of 40.8 to a maximum of 42.3 nm (measurements and error analysis detailed in SI Section S15). No enlargement of the obtained arrays is detected with increased swelling/infiltration times. To confirm these observations and obtain further information on a larger scale (mm^2^), GISAXS experiments were performed on all samples. 2D scattering patterns shown in Figure 6A–D confirm the out-of-plane morphology observed in the SEM micrographs by the presence of intense Bragg rods along *q*_*z*_. Moreover, GISAXS pattern line-cuts along *q*_*y*_ integrated around the Yoneda band (see Figure 6E) confirm no elongation of the nanoarrays with the swelling/infiltration time. Thus, TiO_2_ dots’ *D*_c-c_ distances, calculated with respect to the position of the first Bragg peak (q*),^57^ range between 41.9 to 43.0 nm.

A scatterplot with a line of best fit was made from SEM and GISAXS measurements of *D*_c-c_ (Figure 6F). The line of best fit has a slope of −0.02 nm with an intercept of 41.6 ± 0.4 nm. The slope is effectively zero, confirming there is no change in *D*_c-c_ with respect to time. The intercept can be taken as the *D*_c-c_ of the resulting nanodots from the VPP process. ImageJ was used to measure *D*_c-c_ of dots in XTEM images (see SI Secion S16 for details). The *D*_c-c_ recorded by XTEM is excluded from the linear fit due to the limited accuracy of the small sample size used in the analysis (SI Section S16). There is a higher variance in the *D*_c-c._ Still, the data supports the assertion that there is no increase in *D*_c-c_ with respect to time, as XTEM measurements of *D*_c-c_ pre and post-UV ozone show no correlation between increased infiltration time and change in *D*_c-c_.

A decrease of the full width at half-maximum (FWHM) of the first Bragg rod with respect to increased infiltration time suggested that with increasing exposure times, *D*_c-c_ becomes more uniform, and pattern defects are reduced (SI Section S17). This may be explained by the statistical likelihood of TTIP encountering PEO domain sites increasing with respect to exposure time. This means that the filling rate of PEO domains may not be entirely uniform, and some PEO domains will have a higher level of infiltration than others at different VPP times. Thus, longer exposure time increases the probability of all domains having the same level of infiltration/saturation by TTIP, improving uniformity.

### 3.8 Chemical Characterization of the Obtained Nanodot and Polymer Removal Process by XPS

**Figure 7:**
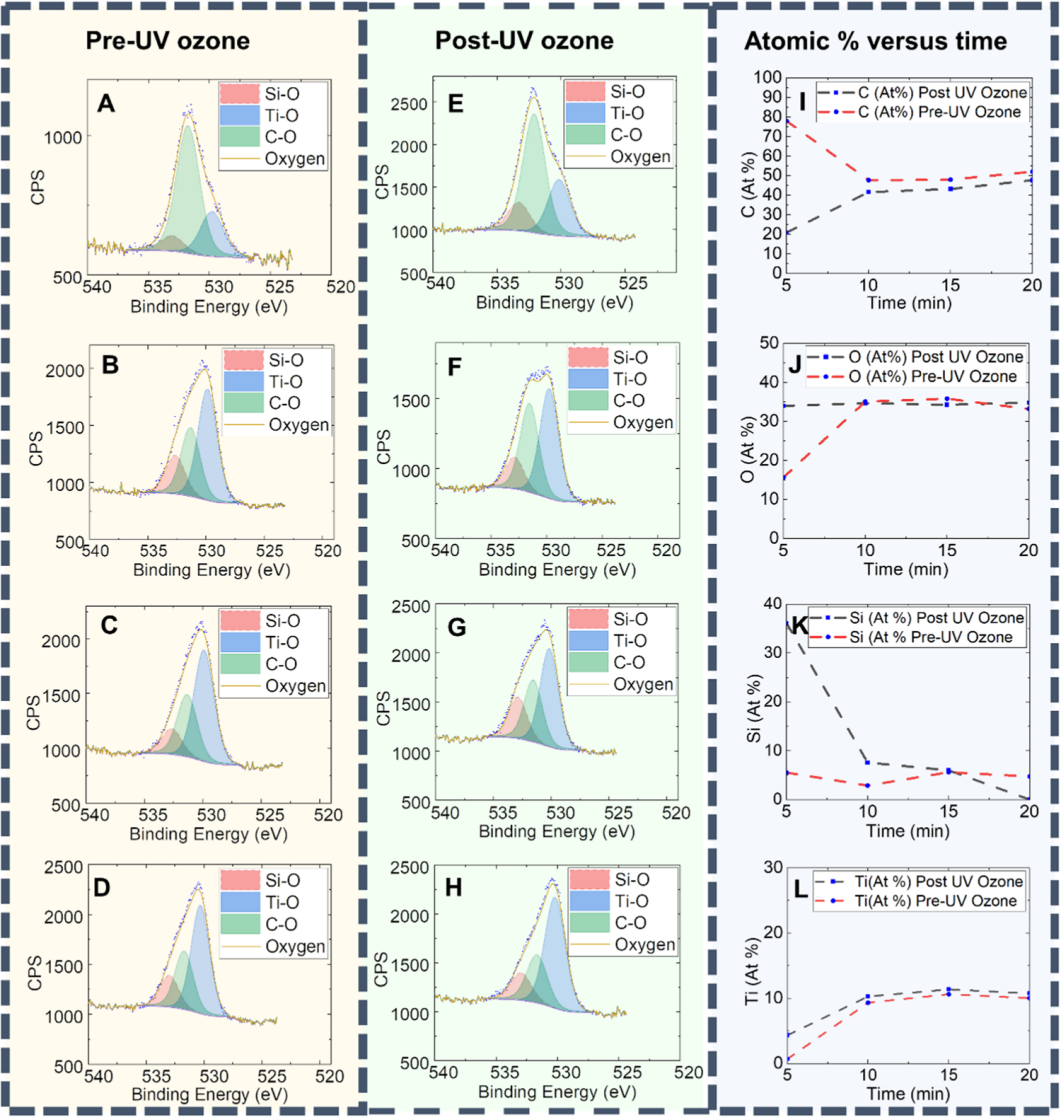
High-resolution XPS spectra for O 1s core levels (A–D) pre-UV ozone and (E–H). post UV-Ozone for exposure times (A, E) 5 min (B, F) 10 min (C, G) 15 min and (D, H) 20 min. Changes in atomic percentage of (I) Carbon, (J) Oxygen, (K) Silicon and (L) titanium pre and post UV-ozone with respect to time.

To investigate the effect of this titanium layer on polymer removal, a comparison of pre-UV ozone peaks to post-UV ozone peaks was done using X-ray photon spectroscopy (XPS). The recorded survey spectra pre- and post-UV ozone confirmed the presence of C and the expected elements (Ti, O, and Si), as shown in SI Figure S12. Binding energies were calibrated by adjusting the adventitious carbon peak positions to 284.8 eV, and the evolution of atomic surface contents is displayed in Figure 7I–L. As depicted in Figure 7, by monitoring the residual carbon in the samples left by UV ozone, the titanium layer above the polymer diminishes the effectivity of the UV ozone polymer removal treatment. Namely, in the case of the 10–20 min exposure time, the C atom % does not decrease as much as expected post-UV ozone due to the capping effect of the TiO_2_ layer (Figure 7I). The 5 min exposure time pre-UV ozone consists of a BCP film with infiltrated PEO domains but without a TTIP film on top, which eventually leads to more polymer removal after oxidation, leading to a larger decrease of C atom % when compared to the 10–25 min exposure samples (Figure 7I).

Thus, with the more efficient polymer removal, more TiO_2_ sites are exposed after UV ozone, leading to a more significant increase in Ti (atom %) (Figure 7L) than in its corresponding pre-UV state. Samples with 10–25 min exposure times have a greater Ti atom % because the titanium layer above the dots increases the surface area of Ti. The increase in Si atom % after UV ozone is greatest for the 5 min exposure time (Figure 7K), which further supports the idea that more polymer is removed when a TiO_2_ film is not present above the dots.

High-resolution spectra were recorded for Ti 2p (SI Figure S13) and O 1s (Figure 7A–H) core levels for the different exposure times. In all cases, pre- and post-UV ozone, the Ti 2p spectra reveal the presence of Ti^4+^ species in TiO_2_ with its characteristic Ti 2p_3/2_ peaks centered at ∼459 eV and a splitting of 5.7 eV in the Ti 2p doublet. The Ti 2p region was fitted keeping constant the peak area ratio between the Ti 2p_1/2_ and Ti 2p_3/2_ spin–orbit coupling splits at 1:2, according to the degeneracy of their spin states. Before and after oxidation, the O 1s spectra consist of a broad peak where 3 local electronic environments can be discerned. The peaks centered at, 533 and 530 eV are attributed to SiO_2_ and TiO_2_, respectively, while the peak at ca. 531.5 eV is ascribed to the BCP (C–O and C=O bonds). Increasing exposure times from 5 to 20 min leads to the growth of the relative contribution of Ti at the surface (Ti–O bonds) revealing a gradual higher degree of TiO_2_ deposition. Comparing pre- and post UV ozone samples, the difference in the relative weight among O species varies in agreement with the polymer removal extent. Namely, while in 15 and 20 min exposure time pre and post UV spectra do not present significant dissimilarities, the relative TiO_2_ contribution increases in the post UV 10 min exposure time as a greater amount of polymer is removed. In the sample subjected to 5 min exposure time, the total O content increases in a large extent after UV ozone, when the polymer removal is more efficient with no TiO_2_ layer deposited atop (Figure 7J).

In summary, the XPS results confirm the formation of TiO_2_ nanodots and the presence of a TiO_2_ film above the nanodots, which form after a 10 min exposure time. This film diminishes the ability of the XPS to detect the BCP template by acting as a capping layer. These observations further support the results in Figure 3.

### 3.9 Toward the Total Structural Control: Future Implementations

The significant potential of the VPP methodology for enabling a highly controlled nanomaterial fabrication process is shown by the control over BCP morphology (Figure 3), feature height (Figure 4C), and feature diameter (Figure 5J). Saturation of the metal domains occurred at a VPP time of 5–10 min because solvent swelling did not uncoil the PEO polymer chains to expose a sufficient number of binding sites for the rate of metal infiltration. With increasing VPP time, the PEO polymer chains continue to uncoil, facilitating increased metal binding and nanodot size; however, a TiO_2_ layer was present on top of the film due to metal oversaturation. Post UV ozone, a Titanium layer with nanodots of different sizes dependent on the VPP time is generated. If producing only nanodots is the study’s objective, then the TTIP flow rate requires further optimization not to saturate PEO domains before sufficient solvent swelling occurs to increase the availability of metal binding sites. Future studies will focus on building an extensive matrix of control variables (temperature, flow rate, time, solvent/metal ratio) that produce uniform nanopatterning with fine feature size and morphology control. Preliminary data are available in SI Section S19, illustrating the effect of improper temperature selection, flow rate, time, and solvent-to-metal ratio. Additionally, a poor choice of a time, temperature, and flow variable can make the system’s kinetics unsuitable for producing high-quality nanopatterning.

A more detailed understanding of the kinetics of the THF and TTIP gas molecules in the chamber is required since the THF and TTIP molecules compete to infiltrate the nanodomains. If alternating cycles of metal precursor infiltration and solvent swelling were used, the system’s kinetics would be simplified. However, as observed during the swelling studies, the film deswells when the flow of the swelling solvent is ceased. Thus, alternating cycles may not sufficiently swell the PEO domains as rapid deswelling could occur between cycles. In this study, dual solvent swelling and metal infiltration hold the PEO chains in a reduced coiled state, exposing more binding sites for increased metal infiltration. The repulsive forces between metal precursor molecules, once bound to the PEO domains, may cause further extension of the chains.^43^ Flory–Huggin’s theoretical framework cannot solely explain the thermodynamics of polymer swelling during dual metal infiltration and solvent swelling processes. New models and, most likely, theoretical simulations are required.

In order to study these effects further and gain better control over the VPP process, we envisage the following improvements: (I) using in situ ellipsometry to monitor film thickness and swell the film to desired feature sizes; this would allow for the determination of the point of metal saturation of the PEO domains. (II) The self-assembly of the PS-*b*-PEO by SVA was done in jars. Even if this technique allows the production of large areas of high-quality nanopatterning, there can be defects due to repeatability issues.^21^ On the contrary, if the SVA annealing is carried out directly in the VPP chamber, BCP films could be immediately held in a swollen state, reducing any chances of pattern defects forming when the film is removed from the SVA process. Moreover, the rate of cooling post-SVA plays a critical role in achieving pattern uniformity, a key requirement for the successful industrial implementation of BCP patterning.^21,22,25,58−61^ (III) The morphology of the BCP template before VPP commences affects the resulting pattern and infiltration time. Metal infiltration into the PEO domains is most rapid in the first 5 min of VPP when infiltration begins with a BCP film of perpendicularly aligned cylinders. After 5 min, when the PS matrix begins to encapsulate the PEO domain and tends toward micelle formation, the rate of infiltration is reduced. This suggests the morphology of the domain before VPP commences affects the infiltration rate. This could be tested in future work by varying the morphology of the initial BCP template and studying the effect on infiltration rate and point of metal saturation. (IV) In this study, we have shown a transition from perpendicularly aligned cylinders to micelles. Future studies should investigate what other changes in morphologies are possible during the VPP process. Finally, (V) combination of UV/O_3_ and calcination could further aid polymer removal and convert the presumably amorphous TiO_2_ to rutile or anaphase crystallographic orientation.^17,37^

## 4 Conclusions

In this study, VPP was used to produce nanodots of different sizes with increased diameter sizes from 20.7 ± 0.1 nm (0 min) to 23.7 ± 0.1 nm (5 min), 25.4 ± 0.1 nm (10 min), 26.9 ± 0.1 nm (15 min), and 28.3 ± 0.1 nm (20 min). VPP can produce nanodots with a 28 nm diameter approximately 15 times faster than the TiO_2_ nanodots produced using ALD by Yin et al.^37^ Recent studies have found that using an additional organic solvent as a coreactant during SIS of polymer films induces solvent swelling of the polymer film and improves the infiltration rate.^40^ However, the necessity to use alternating cycles of precursors limits the extent to which the efficiency of the infiltration rate can be improved. Contrastingly, VPP technology swells the BCP to uncoil polymer domains, increasing the number of accessible reactive sites while infiltrating the nanodomains with metal precursors without alternating cycles. Additionally, dual BCP swelling and metal infiltration allow for manipulation of the original BCP template, increasing pattern uniformity and altering the morphology during infiltration. This capability has not yet been observed for SIS or ALD. The VPP system designed and built for this study has a power consumption similar to that of a regular lab oven. This lowers running costs and may be advantageous for reducing the environmental impacts of nanomanufacturing.

Applications of this nanotechnology are far-reaching. Examples include antibacterial surfaces,^62^ implants such as dental and orthopedic implants,^63,64^ solar cells,^44^ and sensors.^65^ VPP offers a route to defect control, which has been a barrier to integrating BCP patterning into industrial applications where high structural order is required.^10,58^ One such application of VPP is Lithography, an essential cornerstone of the semiconductor industry. Current lithographic techniques are material—and resource-intensive and generate large amounts of toxic waste.^10^ The extension of VPP technology to selectively deposit material without using lithographic masks could hold a promising technological advancement for future semiconductors and other devices. VPP represents a ground-breaking achievement and expands the applicability of BCP patterning in industrial environments.

## Supporting Information Available

ImageJ measurement of feature sizes from XTEM and SEM images; comprehensive error analysis and statistical evaluations of the experimental data are provided; additional information on results obtained using XPS, GISAXS, and ellipsometry; discussion on VPP control variables and potential applications of VPP (PDF)
