# Supplementary material for: Combined Swelling and Metal Infiltration: Advancing Block Copolymer Pattern Control for Nanopatterning Applications
Source: ACS Appl Nano Mater. 2025 Jan 20;8(4):1829–42. doi: 10.1021/acsanm.4c06197 (PMC11791884; doi:10.1021/acsanm.4c06197)
Supplement: Supplementary file 1 — an4c06197_si_001.pdf [file an4c06197_si_001.pdf]

# - Supporting Information -

Combined Swelling and Metal Infiltration: Advancing Block Copolymer Pattern Control for Nanopatterning Applications

Eleanor Mullen [a], Alberto Alvarez-Fernandez [a,b], Nadezda Prochukhan [a], Arantxa Davó-Quiñonero [a,c], Raman Bekarevich [d], Farzan Gity [a,e], Brendan Sheehan [e], Jhonattan Frank Baez Vasquez [a], Riley Gatensby [a], Ahmed Bentaleb [f], Alan Ward g, Paul K. Hurley [a,e] and Michael A. Morris [a*]*

a Centre for Research on Adaptive Nanostructures and Nanodevices (CRANN) and Advanced Materials and Bioengineering Research (AMBER), Trinity College Dublin, Dublin 2, D02 W085, Ireland

b Centro de Física de Materiales (CFM) (CSIC−UPV/EHU) − Materials Physics Center (MPC), Paseo Manuel de Lardizabal 5, 20018 San Sebastián, Spain

c Inorganic Chemistry Department, University of Alicante, Carretera San Vicente del Raspeig s/n, E-03080, Alicante, Spain

d Advanced Microscopy Laboratory (AML), Centre for Research on Adaptive Nanostructures and Nanodevices (CRANN), Trinity College Dublin, Dublin 2, D02 DA31, Ireland

e Tyndall National Institute, University College Cork, Lee Maltings, Cork, T12 R5CP, Ireland

f Centre de Recherche Paul Pascal (CRPP) – UMR 5031, Pessac, 33600 France

g Imperial College London, South Kensington Campus, London SW7 2AZ, United Kingdom

**Corresponding author's email: elmullen@tcd.ie, morrism2@tcd.ie*

Table of Contents

SI Section S1 – In-Situ Ellipsometric Solvent Swelling Studies 3

SI Section S2 - Overview of XTEM ImageJ Analysis 3

SI Section S3 – Solubility Parameters and Solvent Selection 5

SI Section S4 - Solvent Swelling Studies 5

SI Section S5 - Selection of a Suitable Partial Vapor Pressure 6

SI Section S6 - Measurement and Error Analysis of Heights Recorded from XTEM Images 7

SI Section S7 - EDX of XTEM Images 8

SI Section S8 - Nanodot Connections 10

SI Section S9 – Ellipsometry 11

SI Section S10 - Measurement of SEM Images Using ImageJ 11

SI Section S11 - Nanodot Diameter Recorded using ImageJ 12

SI Section S12 - Image Analysis Using Pebbles Software 13

SI Section S13 - Statistical Significance of Gaussian Fittings to Histograms 14

SI Section S14 - Measurement of Diameter from TEM Images 15

SI Section S15 - $D_{c\text{-}c}$ Distance Recorded using ImageJ Processing of SEM Images 17

SI Section S16: Measurement of Dc-c from XTEM Images 18

Section S17 - Decrease of FWHM of the First Bragg Rod with Respect to Time 19

SI Section S18 – XPS 20

SI Section S19 – Importance of Control Variables 21

SI Section S1 – In-Situ Ellipsometric Solvent Swelling Studies

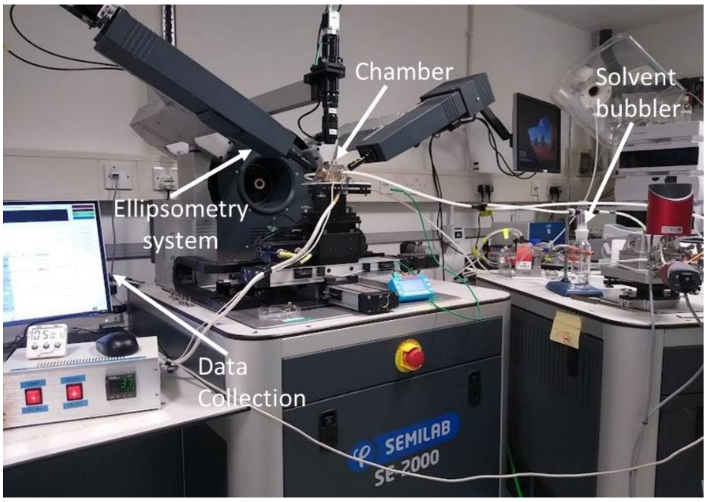

Figure S1 Photograph of the ellipsometry swelling system.

SI Section S2 - Overview of XTEM ImageJ Analysis

The feature sizes of nanodots could not be measured using XTEM images with nanodots that appear in the foreground with an overlap of nanodots in the background as they are indistinguishable (see **Figure S2A**). XTEM Images suitable for recording nanodot height, D_{c-c}, and diameter appear in XTEM **Figure S2B. Figure S2C** shows an XTEM image of the blank PS-*b*-PEO template. The PEO cylinder positions are only visible due to platinum (Pt) infiltration into the PEO domains during electron beam-induced deposition of Pt during FIB. The edges of the PEO cylinders are damaged slightly during the deposition process. Thus, XTEM images of the resultant FIB lamella cannot accurately measure the diameter/width of the PEO domains but serve as an approximation. XTEM images of the PS-*b*-PEO templates can be used for an approximation of Dc-c as the central position of the nanodot is not affected by e-beam damage. Not all PEO domains are thoroughly infiltrated with Pt. Of those fully infiltrated, the recorded thickness offers an approximation of PEO domain thickness.

In the case of BCP templates exposed to VPP, once suitable XTEM images had been collected, feature sizes of the nanodots were recorded using ImageJ. From SEM analysis, we know the nanodots are symmetrical in shape. Thus, if the cross-section for TEM analysis is taken close to the nanodot edge away from the central axis and the beam direction during XTEM is as shown in **Figure S2D**, the resultant TEM image shows a smaller ellipse in a larger ellipse. The actual nanodot diameter and height are not that of the smaller ellipse but that of the larger one, which marks the highest point of the nanodot. If the cross-section is close to the central axis, this is an accurate height measurement due to the low curvature of the top of the nanodot

(**Figure S2E**). Suppose the cross-section for TEM analysis is taken close to the nanodot edge away from the central axis, and the beam direction during XTEM is as shown in **Figure S2F**. Then, the resultant TEM image shows a smaller nanodot segment than the actual nanodot height. It appears as an outlier and can be discounted.

SEM has a lower resolution than TEM, leading to higher variability in SEM measurements, necessitating a larger sample size to reduce the impact of measurement errors. Thus, although XTEM images had a small sample size, the higher resolution allows for higher accuracy cross-sectional heights than SEM. However, other feature size measurements, such as Dc-c and diameter measurements, depend on the angle of the lamella taken. This, combined with the small sample size of these measurements, means XTEM images can approximate the $D_{c\text{-}c}$ and diameter of nanodots. Thus, XTEM is less suitable for these measurements (further details in SI sections **S16 and S14**). SEM is used to record Dc-c and diameter more accurately. A large sample size is used to compensate for the lower resolution of SEM compared to TEM (SI sections **S10).**

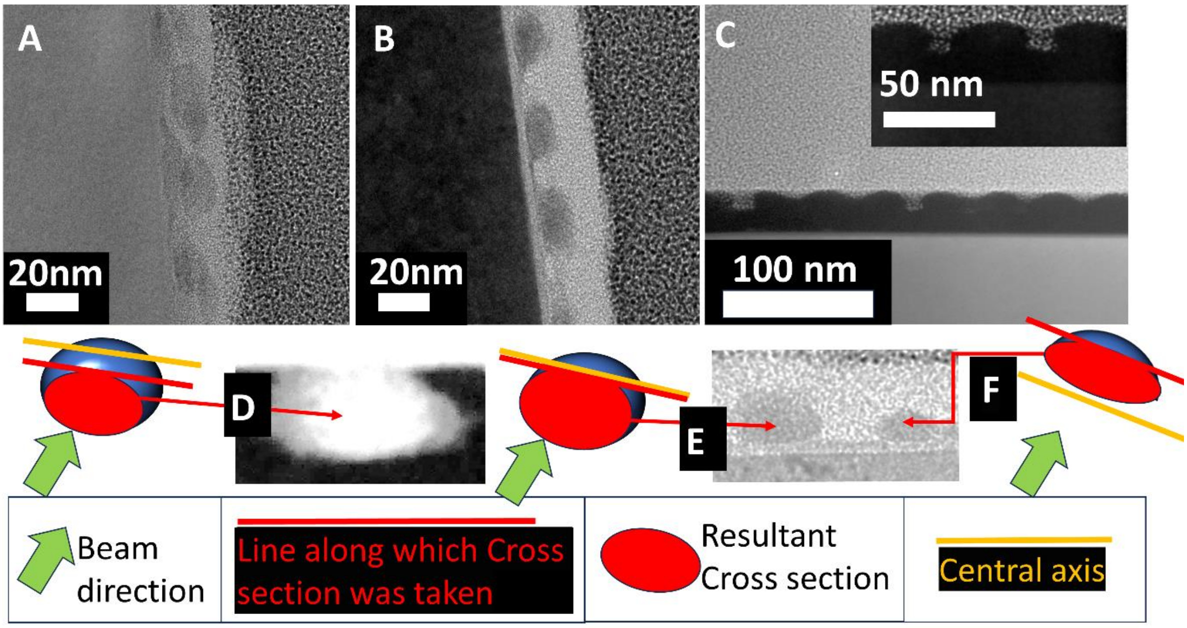

Figure S2 Example of (A) XTEM image that cannot be used to measure nanodot feature sizes. (B) XTEM image is suitable for recording nanodot height, Dc-c, and diameter, and (C) XTEM of PS-b-PEO template. The direction in the cross-sectional segment is taken in, and beam direction affects the recording of nanodot height. In the case of (D), this gives the appearance of two nanodots, one in front of the other; the actual height is the larger nanodot. For (E), the segment direction and beam height give a single nanodot of actual height, and for (F), the perceived nanodot height appears less than the actual height.

SI Section S3 – Solubility Parameters and Solvent Selection

When using the VPP system, the solvent's flow rate during the swelling process, exposure time, choice of temperature, solvent, polymer, and metal precursor determine the nanodots' morphology. The flow rate, temperature, and exposure time are chosen depending on the solvent and metal precursor's vapor pressure and chemical nature. The solvent chosen should not impair the infiltration of the BCP template with the inorganic precursor. Also, the selected solvent should sufficiently swell the BCP template. The Florry-Huggins parameters (χ) were calculated using the Hansen approach for solvent-polymer interactions.[1] For a select solvent, if χ<1, it's a good solvent of the polymer; if $\chi \leq 0.5$, the polymer and solvent are completely miscible and χ>1, it's a bad solvent.[1] The Florry-Huggins parameters are calculated in **Table S1** for THF and Toluene. THF is nonselective and an excellent solvent for both blocks because $X_{EO\text{-}THF}$ and $X_{S\text{-}THF}$ are less than 0.5. Toluene, on the other hand, has an $X_{EO\text{-}Tol}$ >1 and a $X_{S\text{-}Tol}$ <<0.5.[2] Thus, it is only selective for the PS block and does not swell the PEO block.

Table S1 Hansen solubility parameters for PS-*b*-PEO Polyethylene oxide (PEO) and polystyrene (PS) refers to more than one ethylene oxide (EO) and styrene (S); thus, it is the solvent interaction parameter for EO and S that is of interest.

Solvent/polymer	δ_d	δ_p	δ_h	$X_{EO\text{-}B}$	$X_{S\text{-}B}$
THF[2]	16.8	5.7	8	0.27	0.40
Toluene[2]	18	1.4	2	1.85	0.06

SI Section S4 - Solvent Swelling Studies

Equation S1 was used to calculate the percentage increase in film thickness post-swelling, as shown in **Figure 2**, to facilitate comparisons between different films undergoing swelling.

Equation S1 $\left(\frac{Film\ thickness - initial\ thickness}{initial\ film\ thickness}\right) * 100 = Swelling\ (\%)$

The temperature of the toluene swelling process was too low to bring about self-assembly into patterns such as cylinders or gyroids, as observed by Mockarian *et al.*[3] If the BCP in the swollen state is above the order-disorder transition, then rapid solvent removal results in poorly ordered quenched structures. This is likely the case for THF swelling, as the film in **Figure S3E** remains disordered.[4] Thus, regardless of the chosen solvent, self-assembly does not occur if T_S is maintained at 22ºC, T_V is maintained at 18ºC, and the solvent flow rate is 0.1 L/min (**Figure S3** below)

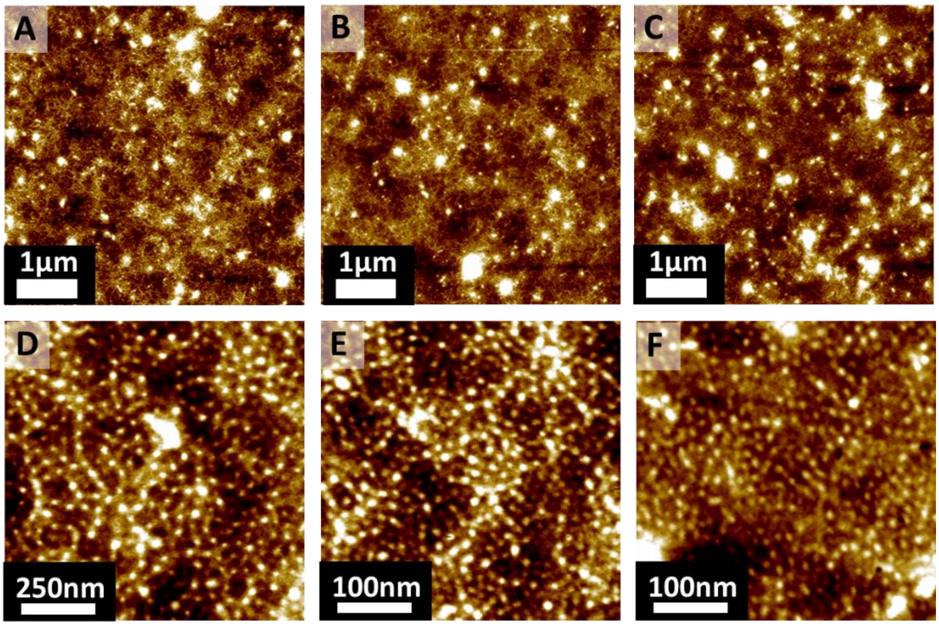

Figure S3 AFM images of BCP films pre and post-solvent swelling. (A and D) Pre-swelling of as-spun BCP film. Swelling of the film with a 0.1 L/min flow rate, T_V of 18°C, T_S of 22°C, and swelling solvent (B and E) THF and (C and F) Toluene.

SI Section S5 - Selection of a Suitable Partial Vapor Pressure

A temperature of 35°C was selected as the optimum temperature to maintain TTIP and THF bubblers during VPP. The vapor pressure of THF is much greater than that of TTIP, thus reducing the probability of TTIP impingement if VPP is performed at high temperatures. Therefore, the theoretical optimum temperature maximizes TTIP saturation pressure and minimizes THF saturation pressure. At 35°C, TTIP vapor pressure increases exponentially with respect to temperature, while THF vapor pressure increases approximately linear with respect to temperature. The saturation pressure was calculated using the Antione equation. Antione parameters for THF and TTIP vapor pressure were obtained from the literature.[5,6]

Thus, the literature suggests an approximate optimum temperature of 35°C for TTIP and THF precursor exposure. However, during the solvent swelling studies, a T_V of 18°C and a T_S of 35°C were trialed. The aim was to determine how the relative difference between T_V and T_S impacts solvent swelling. Based on results from swelling studies, which suggest that the ideal T_S is slightly greater than or equal to T_V, T_V was also set to 35 °C during VPP. Future investigations should further investigate the effect of different temperature choices on VPP in greater detail.

SI Section S6 - Measurement and Error Analysis of Heights Recorded from XTEM Images

If the PEO domains become saturated, the metal precursor is deposited in a thin layer on the BCP template. Thus, for infiltration times greater than or equal to 10 min, the point at which the TiO_2 dots end and the titanium layer begins cannot be distinguished from XTEM images. The layer folds over the TiO_2 dots, taking on their shape. Thus, to measure TiO_2 nanodot height, the combined height of the TiO_2 nanodot and film is calculated and subtracted from the film height measured between the nanodots. The Combined nanodot and film height is measured at the center of the nanodot where height is maximum. For each nanodot or combined nanodot and film height, the ImageJ measurement of height/thickness was repeated 10 times for a single nanodot in an image, and the average was taken. The final height for each VPP exposure time was determined by calculating the average of these individual averages, with the associated error estimated as the standard error of the mean (**SI Table S2**). Film height is measured by taking 50 measurements perpendicularly to the substrate between nanodots. Once again, the error is considered the standard mean error. The error of TiO_2 dot height is the error of the TiO_2 film height plus the error measurement of the combined TiO_2 film and dot height. The recorded average heights and associated errors from pre-UV and post-ozone cross-sectional TEM images are shown in **Tables S2 and S3.**

Table S2 Recorded average heights from cross-sectional TEM images (Post UV ozone for 5-15 min infiltration times and pre-UV ozone for 0min). The associated error is the magnitude of the standard error of the mean.

Time (min)	height (nm)	Feature measured
0	13.3+/-0.4	PEO domain
0	27.9+/-0.1	PS domain
5	11.8+/0.4	nanodots
10	16.4+/-0.5	nanodots + TiO_2 layer
10	4.7 ± 0.2	TiO_2 layer
10	11.7+/- 0.7	nanodots = (nanodots + TiO_2 layer) – TiO_2 layer
15	26.5+/-0.7	nanodots + TiO_2 layer
15	11.1 ± 0.3	TiO_2 layer
15	15.4+/-1	nanodots = (nanodots + TiO_2 layer) – TiO_2 layer

The measurement error of nanodot height produced by a 5 min VPP exposure time is less than a VPP exposure time of 10-30 min because height cannot be measured directly. This is because the titanium layer is not folded over the dots but is deposited on top of the PS layer. The difference between pre and post-UV ozone dot thickness in the case of the 15 min infiltration time is less than the image resolution, so we can assume thickness does not change dramatically post-UV ozone. In the case of the 30 min exposure time, the TiO_2 nanodots are entirely disconnected from the TiO_2. An approximate 2.5+/-0.3nm layer of PS exists between the top of the titanium dot and the TiO_2 film (**Table S3**).

Table S3 Recorded average heights from cross-sectional TEM images (Pre UV ozone). The associated error is the magnitude of the standard error of the mean.

**Time (min)**	**PEO domain with TTIP infiltration**	**PS over PEO**	**Ti layer**
15	14.4 ± 0.3	0	12.5 ± 0.1
30	14.7+/-0.4	2.5+/- 0.3	43.0 ± 0.1

To test for increasing nanodot height with increasing exposure time, linear mixed-effects models were employed, with time as a fixed effect and individual nanodots as a random effect. This approach enabled the variance between repeat measures of the same nanodot to be accounted for while testing for differences between nanodot heights with respect to time. There was a significant increase in nanodot height between 5 and 10 mins (t (df 3.85)= 3.276, p <0.001), with a mean height increasing between 5 and 10 mins from 11.9nm to 16.5. No significant changes in nanodot height were observed after 10 mins. An increase in nanodot height between 5 and 10 min was observed because the PEO domain had not been previously saturated during the 5 min exposure time. At 10 min, the PEO domain is fully saturated, and a titanium layer begins to form above the BCP film, as reported in the main text. In the case of the 30 min exposure time, the nanodot is observed to detach from the titanium layer above the BCP film; however, there is no observed increase in height for this time interval, suggesting that the main effect of increased exposure time is the growth of nanodot diameter.

SI Section S7 - EDX of XTEM Images

Energy dispersive X-ray spectroscopy (EDX) maps for all XTEM images in **Figure 3A** (main text) are shown below in **Figure S4**. **Figure S4A** (5 min) shows nanodot formation with no

TiO_2 on top**. Figure S4B** (10 min) and **Figure S4C** (15 min) show the formation of a thin layer of TiO_2 on top of the nanodots and over the PS domains. **Figure S4D** and **Figure S4E** illustrate how a VPP time increase from 15 to 30 min causes micelle formation. **Figure S4F** is an XTEM image of the PS-*b*-PEO template with two scan areas marked on the image. Scan 1 (**Figure S4G**) has a Pt peak because Pt has infiltrated the PEO domains marked in **Figure S4F**. Scan 2 (**Figure S4H**) has no Pt peaks because the polymer layer has not accepted metal ions. PEO has reactive sites for metal binding, whereas PS does not.

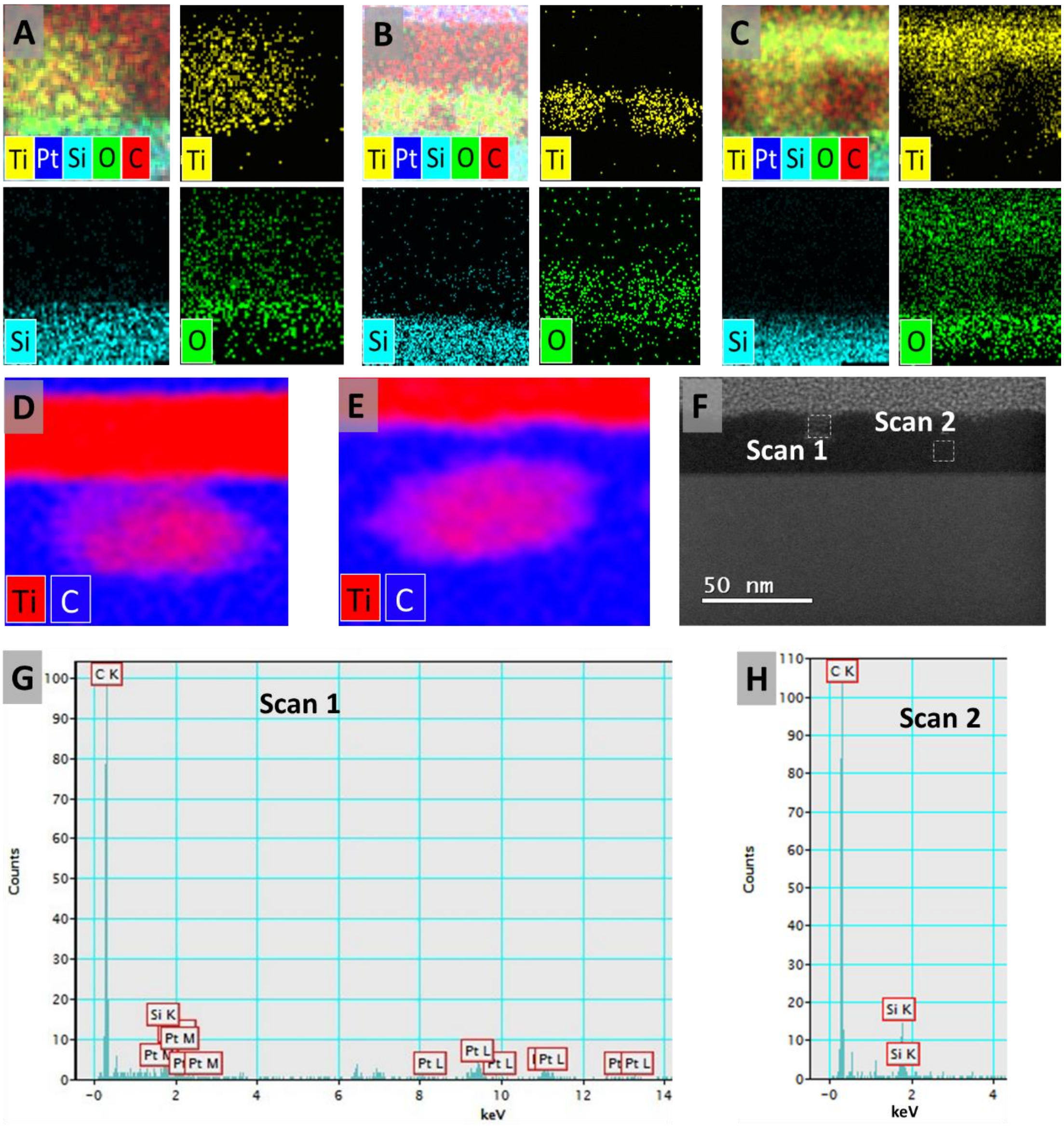

Figure S4 EDX images corresponding to **Figure 3A** in the main text. Post UV ozone images of VPP times (A) 5 min, (B) 10 min, and (C) 15 min are shown in the top row. Pre-UV ozone images of (D) 15 min, (E) 30 min, and (F) 0 min are shown in the middle row. (G) and (F) are EDX maps of areas marked scan 1 and scan 2 in (F).

SI Section S8 - Nanodot Connections

If a 5 min VPP time is used, the SEM (**Figure S5A**) and TEM (**SI Figure S5B**) images show that thin TiO2 nanowires connect some nanodots. One possible mechanism for the formation of nanowires between nanodots is the saturation of PEO domains with TTIP precursor, which leads to TTIP nucleation and growth between nanodots along defects in the self-assembled pattern. These defects can be attributed to non-uniform domain orientation due to unfavorable interfacial interactions of the BCP domain and substrate. The appearance of nanowire structures between nanodots provides the potential for applications such as electronics.[7] However, controlling those defects in future work would be beneficial in producing a uniform network of connected nanodots. One means of removing template defects is to provide a matching surface template for surface neutrality to eliminate the effect of the substrate's surface effects, such as using a polymer brush to promote improved orientation of the polymer block and to improve the uniformity of the metal deposition.[8] Multilevel molecular self-assembly could then be used to precisely direct the patterning of TiO2 nanowires on top of previously patterned arrays of nanodots.[9] Alternatively, methods, such as scanning probe lithography, could be used to etch lines between nanodots to promote the growth of TTIP nucleation between nanodots along the etched line.

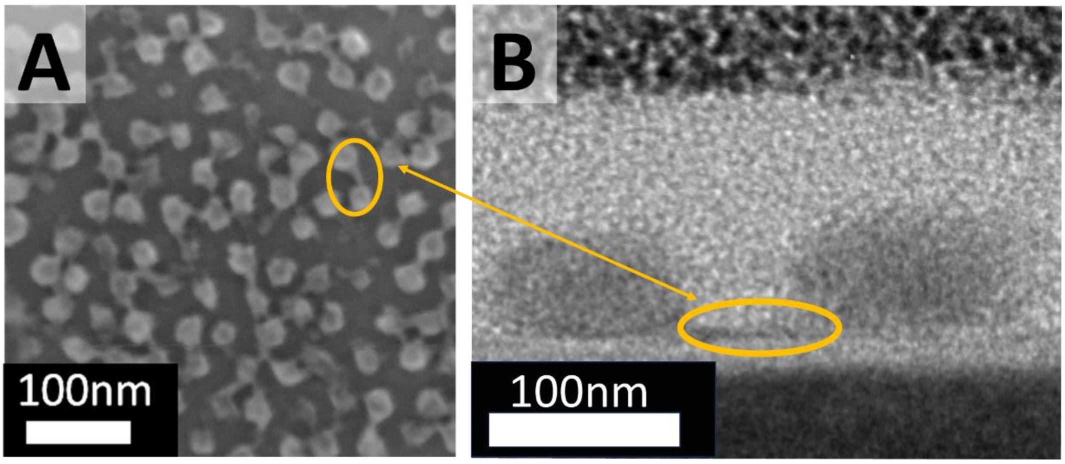

Figure S5 (A) topographical SEM image and (B) XTEM image illustrating the presence of TiO_2 nanowires.

SI Section S9 – Ellipsometry

Table S4 Ellipsometry thicknesses post UV ozone.

Time (min)	Thickness Post-UV Ozone (nm)	Pattern
5	12.7	Nanodots
10	16.8	Nanodots
15	25.6	Nanodots
20	28.4	Nanodots

SI Section S10 - Measurement of SEM Images Using ImageJ

All SEM images analyzed were of Post UV ozone samples. SEM images were imported into ImageJ and the scale set. The contrast/greyscale was not adjusted for both $D_{c\text{-}c}$ and diameter measurements. The recording of feature sizes of nanodots began from the bottom left of each image and ended at the top right (**illustrated in Figures S6A and S6B**. Once all nanodots were measured, nanodots were re-measured in a random order until a sample size of 500 nanodots was recorded. If $D_{c\text{-}c}$ was being measured, the following procedure is used: Two concentric circles are superimposed onto every nanodot in the SEM image for a particular VPP exposure time. The inner concentric circle has a diameter tending towards zero. The outer concentric circle has a diameter equal to the average nanodot diameter for the VPP exposure time being studied. The $D_{c\text{-}c}$ is measured between the inner concentric circles of two neighboring nanodots (**illustrated in Figure S6C and S6D**). Measurements began with the distance between the center of nanodots in row 1 and row 2 (**Figure S6C**). Subsequently, progressing to the adjacent row $D_{c\text{-}c}$ distance was measured between nanodots along row 2 and row 3 (**Figure S6D**). This procedure was iterated until measurements were taken across all rows.

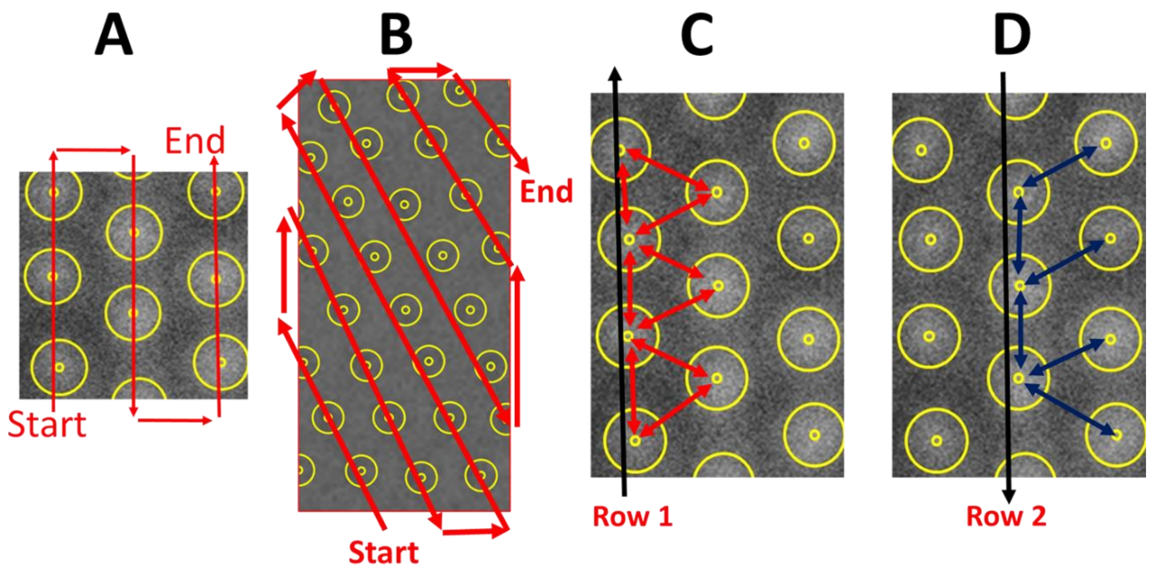

Figure S6 Diagrammatic steps for measuring $D_{c\text{-}c}$ and diameter using ImageJ. (A) and (B) illustrate how measurements are recorded along a line of nanodots moving from bottom left to top right. (C) and (D) explain the order in which $D_{c\text{-}c}$ is measured

SI Section S11 - Nanodot Diameter Recorded using ImageJ

The diameters recorded using ImageJ analysis are shown in **Table S5**. The standard error of the mean is taken to be the measurement error and thus is used for the error bars in **Figure 5J** (main text). The measurement error is less than the resolution of the SEM.

Table S5 Diameter and statistical analysis from ImageJ measurements of different exposure times.

Time (mins)	Diameter (nm)	Standard error of the mean (nm)
0	20.7	0.1
5	23.7	0.1
10	25.4	0.1
15	26.9	0.1
20	28.3	0.1

SI Section S12 - Image Analysis Using Pebbles Software

This section details the analysis of feature sizes using Pebbles software.[10] The analysis method done in ImageJ did not require grey scale/contrast changes. Contrastingly, the greyscale/contrast of the SEM images required adjustment in ImageJ before being imported into Pebbles as TIFF files. This is because automatic mode was used to record the diameter of the nanodots, and contrast had to be sufficiently high for software recognition of the nanodots.[11] However, changing the image contrast can impact the reliability of nanodot recognition software. The areas scanned depended on the magnification of the SEM image and ranged from 0.25μm^2-0.49 μm^2. The diameter of the obtained nanofeatures grows with increased swelling/infiltration time (**Table S6**). SEM images in **Figure S7** correspond to (A) 0 min, (B) 5 min, (C) 10 min, (D) 15 min, and (E) 20 min VPP times. The Pebbles software overlays SEM images with circles of varying diameters, which depend on the boundaries the software recognizes as the nanodots (**Figure S7F-J**). **Figure S7K** shows histograms of the recorded data.

Table S6 Diameter and statistical analysis from Pebbles measurements of different exposure times

Time (mins)	Diameter (nm)	Standard error of the mean (nm)
0	22.5	0.1
5	23.8	0.1
10	26.1	0.1
15	30.9	0.1
20	32.9	0.1

Diameters recorded by ImageJ analysis of SEM and XTEM images and Pebbles analysis of SEM images are plotted in **Figure S7L**. SEM images for samples Post UV ozone for 15 and 20 min VPP times required higher contrast and grey scale adjustments compared to 0-10min VPP times. This affected the accuracy of the pebbles software, which overapproximated the diameter of 15 and 20 min VPP times. This suggests that ImageJ is a more suitable approach. Furthermore, measurements recorded by ImageJ analysis of SEM analysis are in closer agreement with the XTEM data. However, due to the minimal sample size and the dependence of observed diameters on the angle at which the lamella is taken, XTEM data had

a lower accuracy than SEM (**SI Section S2**). Thus, XTEM can support the observed trend in SEM data but needs a sufficiently large sample size to record diameter accurately.

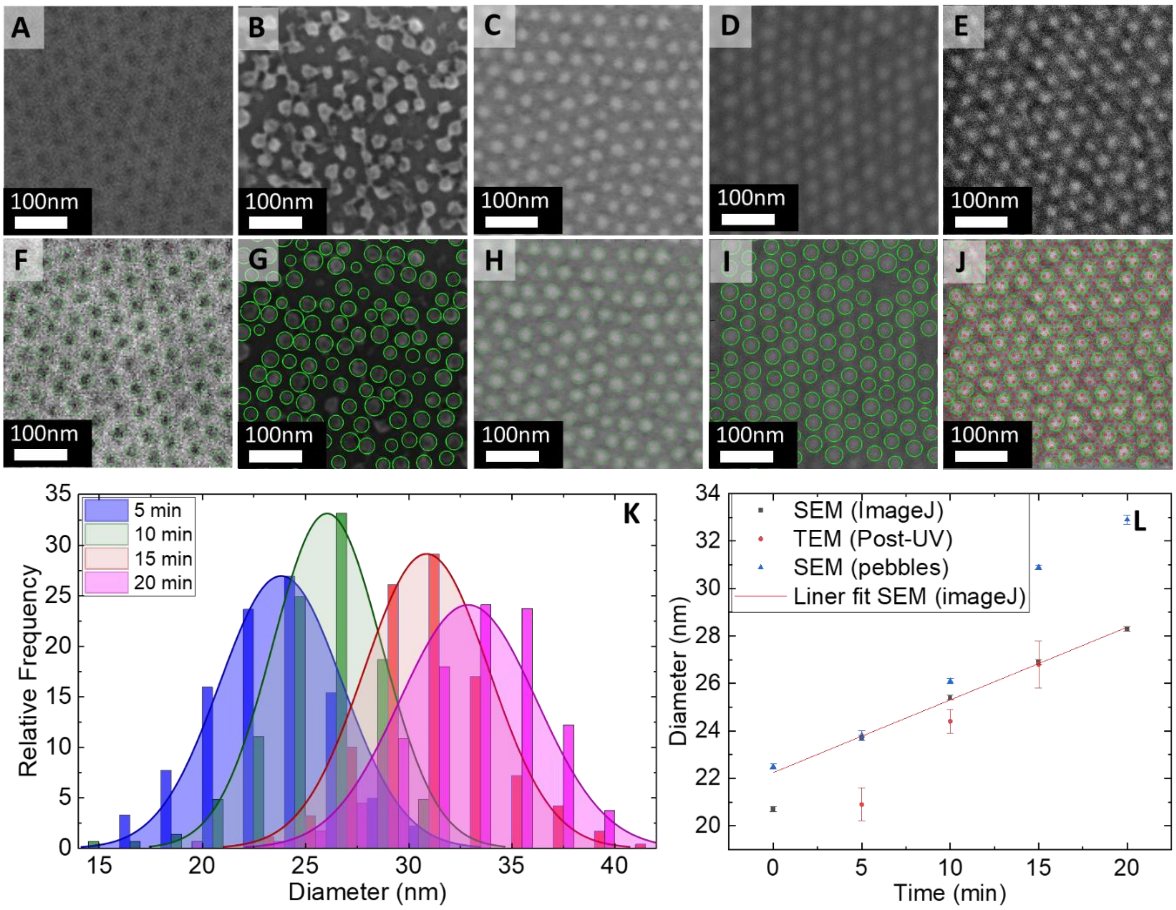

Figure S7 Top-view SEM micrographs of the TiO_2 nanodots obtained after different SVA/infiltration times: (A) 0 min, (B) 5 min, (C) 10 min, (D) 15 min, and (E) 20 min. (F–J) Image overlay with Pebbles fitting of diameter. (K) Corresponding nanodot diameter histograms obtained by Pebbles image analysis. (L) Plot of the average diameter recorded for different exposure times using SEM and XTEM analysis

SI Section S13 - Statistical Significance of Gaussian Fittings to Histograms

A linear model was used to evaluate the increase in nanodot diameter with time, with a post-hoc TUKEY HSD test conducted to test for pairwise differences between nanodot diameter and each time category. There was a significant increase in nanodot size with each increase in the time interval (F_{2495}= 440.7, $P<0.05$ in the case of analysis using ImageJ and F_{1147}= 427.3, $P<0.05$ in the case of analysis using Pebbles). In the case of ImageJ analysis, effect sizes of 10-5 min = 1.7 nm, 15-10 min = 1.5 nm, and 20-15 min = 1.4 nm were recorded. The effect size was significant for each successive time ($P<0.05$)). In the case of Pebbles software

analysis, effect sizes of 10-5 min = 2.3 nm, 15-10 min = 4.8 nm, and 20-15 min = 2nm were recorded. Once again, the effect size was significant for each successive time ($P<0.05$)).

The GISAXS data suggest that nanodot sizes become more uniform and defects are reduced as exposure times increase. The nanodot diameter histograms obtained from SEM images on the nanoscale are less accurate than wide-area micro scans done using GISAXS. Thus, we still conclude that nanodot sizes become more uniform, and defects are reduced as exposure times increase.

SI Section S14 - Measurement of Diameter from TEM Images

XTEM analysis of diameter size confirmed that nanodot diameter increases with respect to time and supports the trend discussed in **SI Section 13**. However, the diameters recorded have limited accuracy due to the small sample size. Additionally, nanodot size is affected by the angle at which the cross-section was taken relative to the hexagonal pattern of the nanodots. Suppose the cross-section cuts through the front of the nanodot. In that case, the TEM image will give the illusion of a smaller ellipse within a larger ellipse, as shown in **Figure S8A**. The smaller ellipse is simply a result of the removal of part of the nanodot. The actual diameter can only be recorded along the major axis of the larger ellipse, as shown in the XTEM image in **Figure S8B**. If the angle of the lamella taken included the three nanodots, as shown in **Figure S8C** then the XTEM image will show two brighter nanodots with a darker nanodot in between. The actual diameter of the nanodots in the foreground is then measured along the central axis of the brighter ellipse.

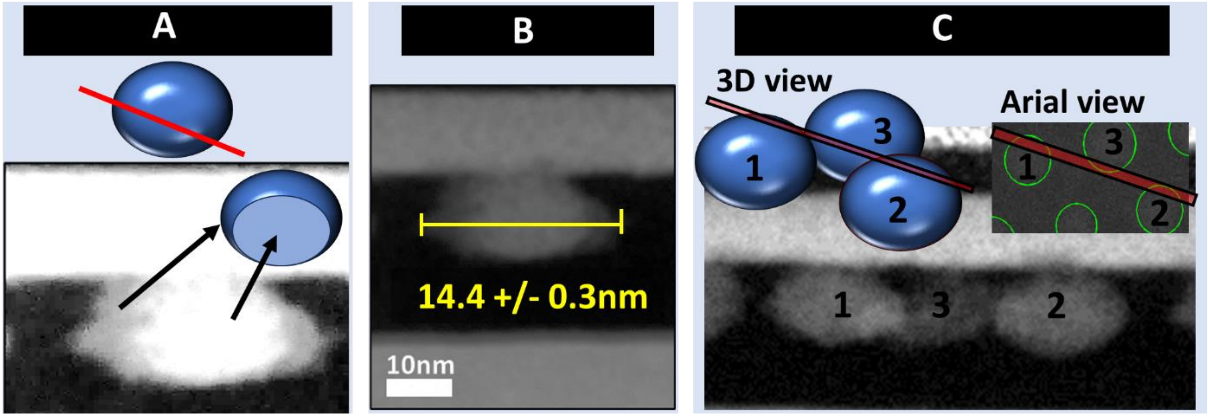

Figure S8 Limitations of measuring diameter using XTEM images. (A) Effect of lamella cutting through nanodot off central axis (B) True diameter is measured at widest points of nanodot (C) Nanodots may overlap if lamella is taken in the position shown by the red line.

For each nanodot, the ImageJ diameter measurement was repeated 10 times for a single nanodot in an image, and the average was taken. The final diameter value for each VPP exposure time was determined by calculating the average of these individual averages, with the associated error estimated as the standard error of the mean. Results are shown in **Table S7** and **Table S8.**

Table S7 Recorded average diameters from cross-sectional TEM images (post-UV ozone). The associated error is the magnitude of the standard error of the mean.

Time (min)	Diameter (nm)
5	20.9+/-0.7
10	24.4+/-0.5
15	26.8+/- 1

The diameter measurement at 0 min in the BCP template provides a rough approximation (**Table S8**). Very few PEO domains had sufficient Pt infiltration during the FIB process for high enough measurement contrast, meaning the sample size was much less than all other recorded diameters. Thus, this measurement is excluded from **Figure 5J** (main text).

Table S8 Recorded average diameters from cross-sectional TEM images (pre-UV ozone). The associated error is the magnitude of the standard error of the mean.

Time (min)	Diameter (nm)
0	21.5 ± 1.2
15	25.7 ± 0.7
30	28.8 ± 1.0

To test for increasing nanodot diameter with increasing exposure time, linear mixed-effects models were employed, with time as a fixed effect and individual nanodots as a random effect. This approach enabled the variance between repeat measures of the same nanodot to be accounted for while testing for differences between nanodot diameters with respect to time. There was a significant increase in nanodot diameter with increased time (t (df 16.85)=33.77, $p < 0.005$), with an increase of ~2.6nm between time bands. Nanodot diameter, as measured with TEM, increased significantly with time. Thus, despite the minimal sample size and the dependence of observed diameters from XTEM analysis, it still offers a means of confirming the trend of nanodot diameter increasing with respect to time.

SI Section S15 - D_{c-c} Distance Recorded using ImageJ Processing of SEM Images

The SEM images in **Figure S9A-D** and **Figure S9I** are Post-UV and pre-ozone SEM images of nanodots with VPP infiltration times of 0, 5, 10, 15, and 20 min. Concentric circles are superimposed onto the SEM images in **Figure S9E-H** and **Figure S9J**. The outer width of these circles is the average nanodot diameter recorded for the particular infiltration time. A histogram of the D_{c-c} was plotted in **Figure S9K** using the results from ImageJ analysis D_{c-c} for the different VPP exposure times. The histograms almost completely overlap as the mean D_{c-c} for each VPP exposure time are very close (as seen in **Table S9**). The standard error of the mean is taken to be the measurement error and thus is used for the error bars in **Figure 6F** (main text).

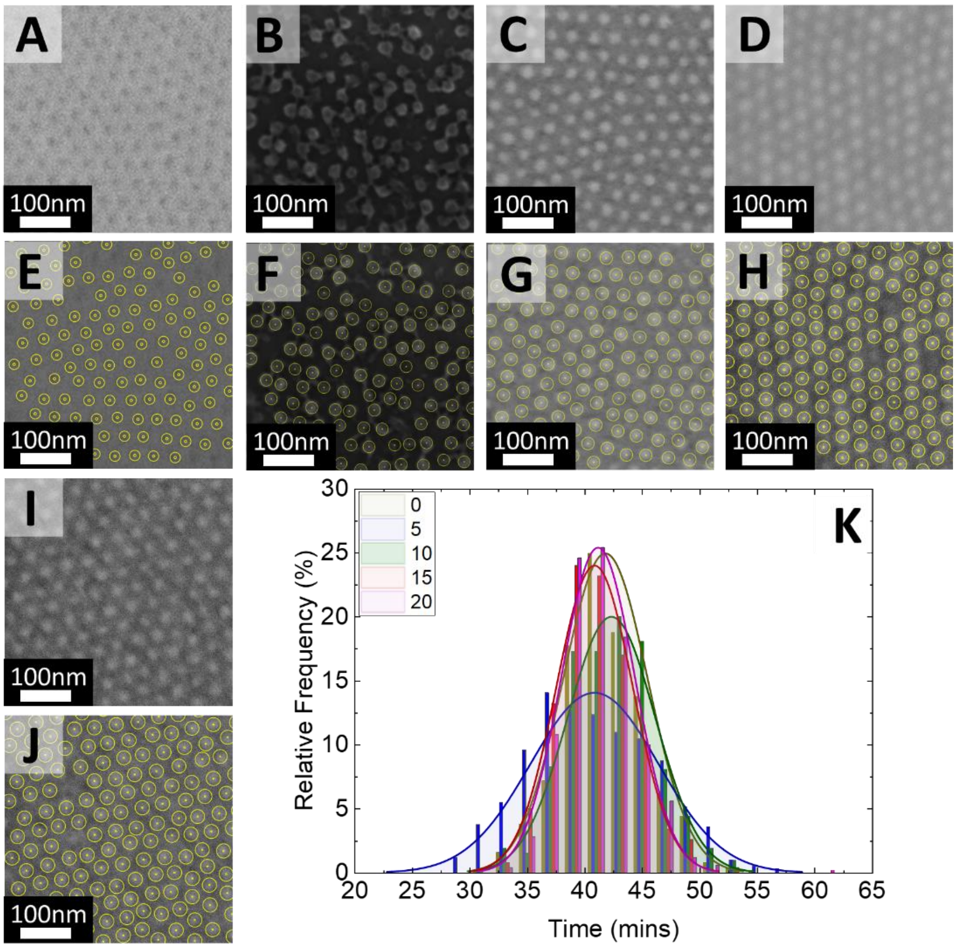

Figure S9 – SEM images of infiltration times (A) 0 min, (B) 5 min, (C) 10 min, (D) 15 min, and (I) 20 min. Concentric circles overlayed on top of nanodots with a diameter corresponding to infiltration times of (E) 0 min, (F) 5 min, (G) 10 min, (H) 15 min, and (J) 20 min. (K) Histograms were obtained from D_{c-c} measurements using ImageJ analysis of images (E-H) and (J).

Table S9 D_{c-c} recorded using ImageJ

Time (mins)	D_{c-c} (nm)	Standard error of the mean (nm)
0	41.8	0.2
5	40.8	0.2
10	42.3	0.2
15	40.9	0.2
20	41.2	0.1

SI Section S16: Measurement of Dc-c from XTEM Images

The illustration in **Figure S10A** shows how, depending on whether the lamella is taken in the orientation of line 1 or 2, the nanodots will appear closer together or further apart, given the hexagonal arrangement of nanodots. Thus, results from any measurement of D_{c-c} are limited as the result varies dramatically depending on lamella orientation. This is further illustrated by the XTEM image of nanodots from a 5 min VPP process in **Figure S10B**. The distance between the nanodots is not constant due to the dependence on the orientation in which the lamella is taken. The ImageJ measurement of the distance between a single pair of nanodots was repeated 10 times, and the average was taken. This was done for all D_{c-c}. The final D_{c-c} value for each VPP exposure time was determined by calculating the average of these individual averages, with the associated error estimated as the standard error of the mean (**Table S10 and S11**).

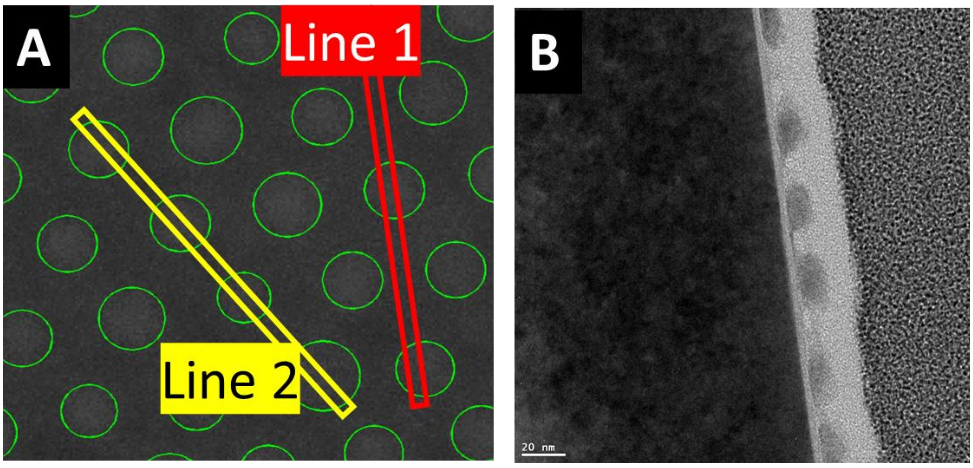

Figure S10 The variability of D_{c-c} measured by TEM. (A) Orientation of lamella effects D_{c-c} in XTEM images such as in (B). (B) XTEM image of a 5 min VPP.

Table S10 Recorded Dc-c from XTEM images (post-UV-ozone). The associated error is the magnitude of the standard error of the mean.

**Time (min)**	**Dc-c (nm)**
5	34.2 ± 2.5
10	38.3 ± 1.4
15	39.0 ± 0.7

Table S11 Recorded Dc-c from XTEM images (post-UV-ozone). The associated error is the magnitude of the standard error of the mean.

**Time (min)**	**Dc-c (nm)**
0	38.8 ± 0.6
15	39.0 ± 0.7
30	35.2 ± 0.9

Section S17 - Decrease of FWHM of the First Bragg Rod with Respect to Time

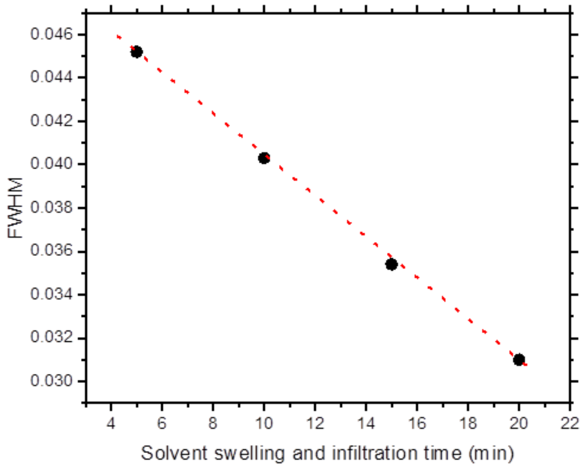

Figure S11 Graph showing the effect of increased solvent swelling and infiltration time on FWHM of the first Bragg rod.

SI Section S18 – XPS

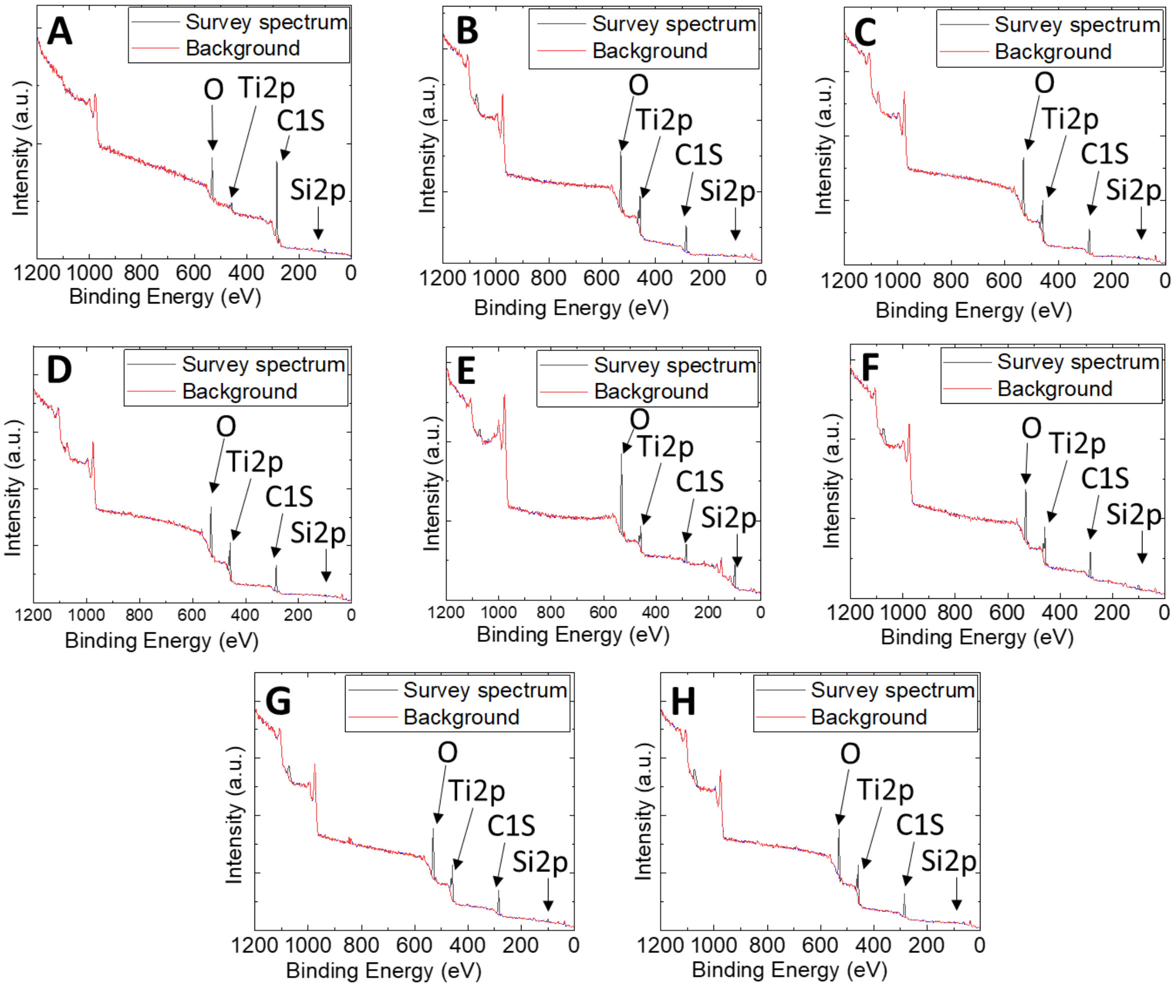

Figure S12 Survey spectra of exposure times (A, E) 5min (B, F) 10min (C, G) 15min and (D, H) 20min both (A-D) pre-UV Ozone and (F-G) post UV ozone.

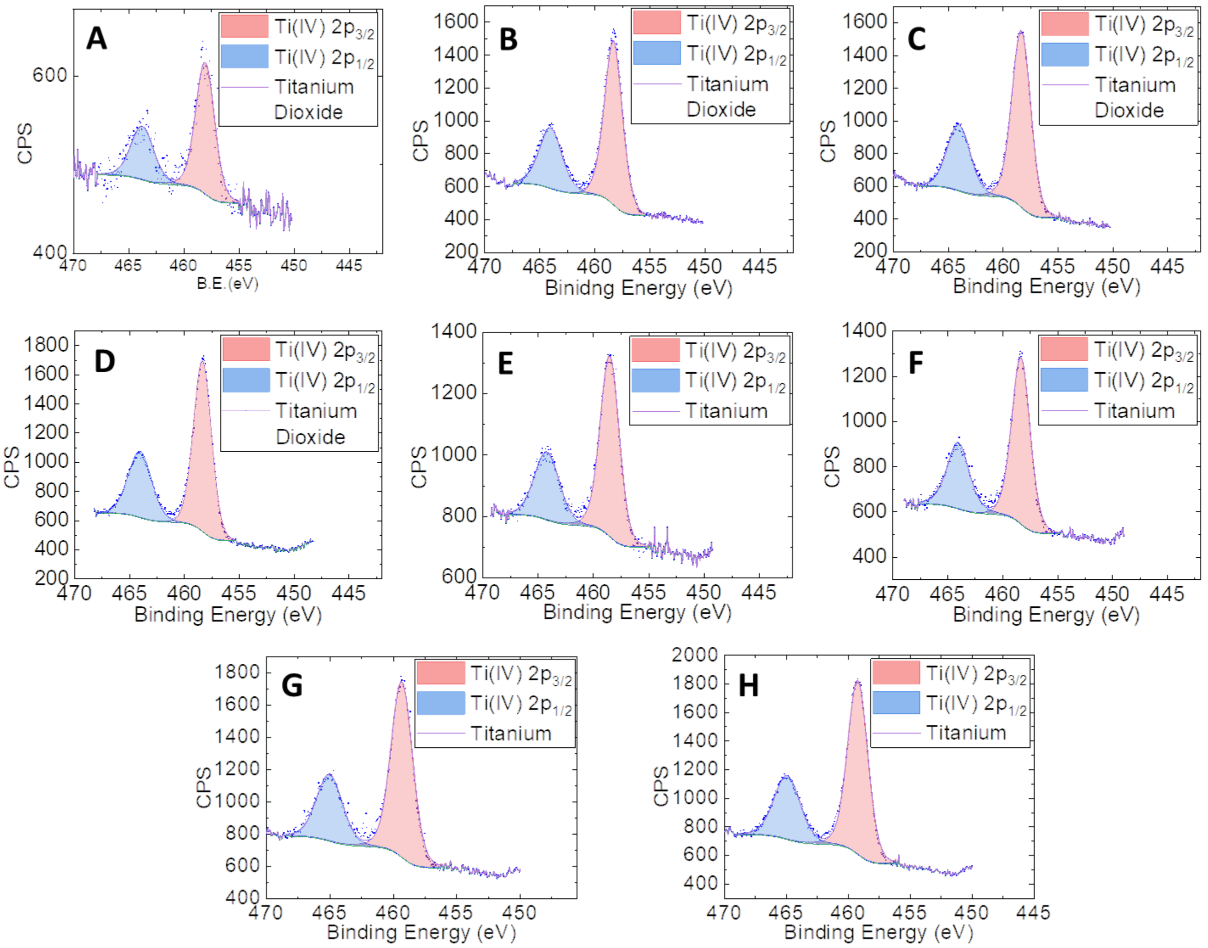

Figure S13 High-resolution XPS spectra for Ti 2p core levels (A-D) pre-UV ozone and (E-H) post-UV Ozone for exposure times (A, E) 5min (B, F) 10min (C, G) 15min and (D, H) 20min.

SI Section S19 – Importance of Control Variables

The correct balance between flow rate, exposure time, metal precursor: solvent ratio and temperature is central to achieving high-quality pattern control. **Figure S14**A-B shows a BCP film of PS(25K)-*b*-P4VP(10K) that was first self-assembled into vertical cylindrical arrays and then placed in the VPP system. Post-VPP patterning is lost, and the film is swollen. In this case, the selection of control variables requires further optimization.

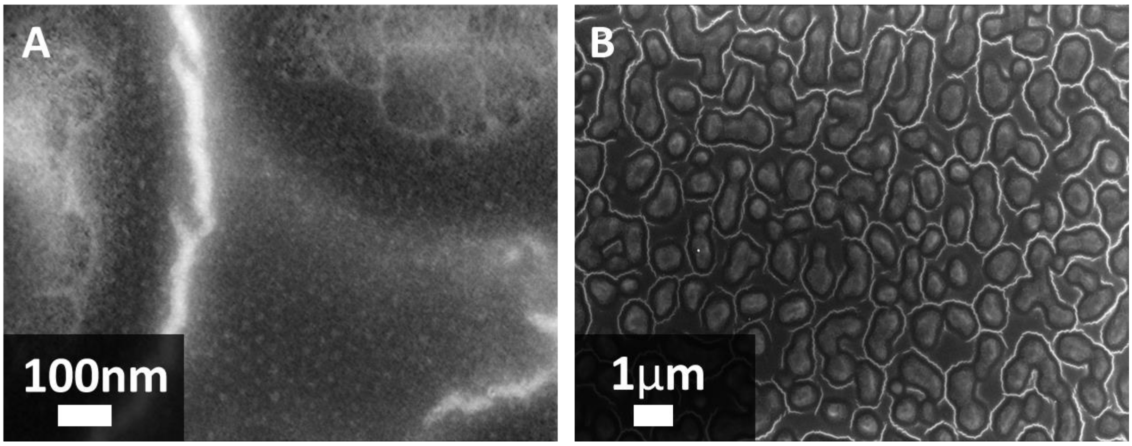

Figure S14 SEM images of PS(25K)-b-P4VP(10K) post VPP with (A) 100nm scale bar and (B) a 1µm scale bar.

References:

(1) Lindvig, T.; Michelsen, M. L.; Kontogeorgis, G. M. A Flory-Huggins Model Based on the Hansen Solubility Parameters. *Fluid Phase Equilib* **2002**, *203* (1–2), 247–260. https://doi.org/10.1016/S0378-3812(02)00184-X.

(2) Alvarez-Fernandez, A.; Fornerod, M. J.; Reid, B.; Guldin, S. Solvent Vapor Annealing for Controlled Pore Expansion of Block Copolymer-Assembled Inorganic Mesoporous Films. *Langmuir* **2022**, *38* (10), 3297–3304. https://doi.org/10.1021/acs.langmuir.2c00074.

(3) Mokarian-Tabari, P.; Collins, T. W.; Holmes, J. D.; Morris, M. A. Cyclical "Flipping" of Morphology in Block Copolymer Thin Films. *ACS Nano* **2011**, *5* (6), 4617–4623. https://doi.org/10.1021/nn2003629.

(4) Hanley, K. J.; Lodge, T. P.; Huang, C. I. Phase Behavior of a Block Copolymer in Solvents of Varying Selectivity. *Macromolecules* **2000**, *33* (16), 5918–5931. https://doi.org/10.1021/ma000318b.

(5) Christau, S.; Thurandt, S.; Yenice, Z.; von Klitzing, R. Stimuli-Responsive Polyelectrolyte Brushes as a Matrix for the Attachment of Gold Nanoparticles: The Effect of Brush Thickness on Particle Distribution. *Polymers (Basel)* **2014**, *6* (7), 1877–1896. https://doi.org/10.3390/polym6071877.

(6) Keller, A.; Wlokas, I.; Kohns, M.; Hasse, H. Thermophysical Properties of Mixtures of Titanium(IV) Isopropoxide (TTIP) and p-Xylene. *J Chem Eng Data* **2020**, *65* (2), 869–876. https://doi.org/10.1021/acs.jced.9b01059.

(7) Nickerson, N. H.; Li, Y.; Benjamin, S. C. Topological Quantum Computing with a Very Noisy Network and Local Error Rates Approaching One Percent. *Nat Commun* **2013**, *4*, 1–5. https://doi.org/10.1038/ncomms2773.

(8) Prochukhan, N.; Selkirk, A.; Lundy, R.; Giraud, E. C.; Ghoshal, T.; Downing, C.; Morris, M. A. Large-Area Fabrication of Vertical Silicon Nanotube Arrays via Toroidal Micelle Self-Assembly. *Langmuir* **2021**, *37* (5), 1932–1940. https://doi.org/10.1021/acs.langmuir.0c03431.

(9) Shin, D. O.; Mun, J. H.; Hwang, G. T.; Yoon, J. M.; Kim, J. Y.; Yun, J. M.; Yang, Y. B.; Oh, Y.; Lee, J. Y.; Shin, J.; Lee, K. J.; Park, S.; Kim, J. U.; Kim, S. O. Multicomponent Nanopatterns by Directed Block Copolymer Self-Assembly. *ACS Nano* **2013**, *7* (10), 8899–8907. https://doi.org/10.1021/nn403379k.

(10) Mondini, S.; Ferretti, A. M.; Puglisi, A.; Ponti, A. Pebbles and PebbleJuggler: Software for Accurate, Unbiased, and Fast Measurement and Analysis of Nanoparticle Morphology from Transmission Electron Microscopy (TEM) Micrographs. *Nanoscale* **2012**, *4* (17), 5356–5372. https://doi.org/10.1039/c2nr31276j.

(11) Mondini, S.; Ferretti, A. M.; Puglisi, A.; Ponti, A. Pebbles and PebbleJuggler: Software for Accurate, Unbiased, and Fast Measurement and Analysis of Nanoparticle Morphology from Transmission Electron Microscopy (TEM) Micrographs. *Nanoscale* **2012**, *4* (17), 5356–5372. https://doi.org/10.1039/c2nr31276j.
